# Author Correction: Deficiency of Axl aggravates pulmonary arterial hypertension via BMPR2

**DOI:** 10.1038/s42003-022-03045-0

**Published:** 2022-01-20

**Authors:** Tatyana Novoyatleva, Nabham Rai, Baktybek Kojonazarov, Swathi Veeroju, Isabel Ben-Batalla, Paola Caruso, Mazen Shihan, Nadine Presser, Elsa Götz, Carina Lepper, Sebastian Herpel, Grégoire Manaud, Frédéric Perros, Henning Gall, Hossein Ardeschir Ghofrani, Norbert Weissmann, Friedrich Grimminger, John Wharton, Martin Wilkins, Paul D. Upton, Sonja Loges, Nicholas W. Morrell, Werner Seeger, Ralph T. Schermuly

**Affiliations:** 1 Universities of Giessen and Marburg Lung Center (UGMLC), Excellence Cluster Cardio-Pulmonary System (ECCPS), Member of the German Center for Lung Research (DZL), Justus-Liebig-University Giessen, Giessen, Germany; 2 Institute for Lung Health, Giessen, Germany; 3 Department of Oncology, Hematology and Bone Marrow Transplantation with section Pneumology, Hubertus Wald University Comprehensive Cancer Center Hamburg, University Medical Center Hamburg-Eppendorf, Hamburg, Germany; 4 Department of Tumor Biology, Center of Experimental Medicine, University Medical Center Hamburg-Eppendorf, Hamburg, Germany; 5 Department of Medicine, University of Cambridge, Cambridge, UK; 6 AP-HP, INSERM UMR_S 999, Service de Pneumologie et Soins Intensifs Respiratoires, Hôpital de Bicêtre, Université Paris–Saclay, Le Kremlin Bicêtre, France; 7 Centre for Pharmacology and Therapeutics, Department of Medicine, Imperial College London, London, UK; 8 Max Planck Institute for Heart and Lung Research, Bad Nauheim, Germany

**Keywords:** Growth factor signalling, Apoptosis, Cardiac hypertrophy

Correction to: *Communications Biology* 10.1038/s42003-021-02531-1, published online 24 Aug 2021.

In the original version of the Article, Fig. 4a mistakenly included a surplus lane in the Pan-actin loading control western blot. This has now been corrected, displaying 14 lanes across the panel.

The correct version of Fig. 4 is:
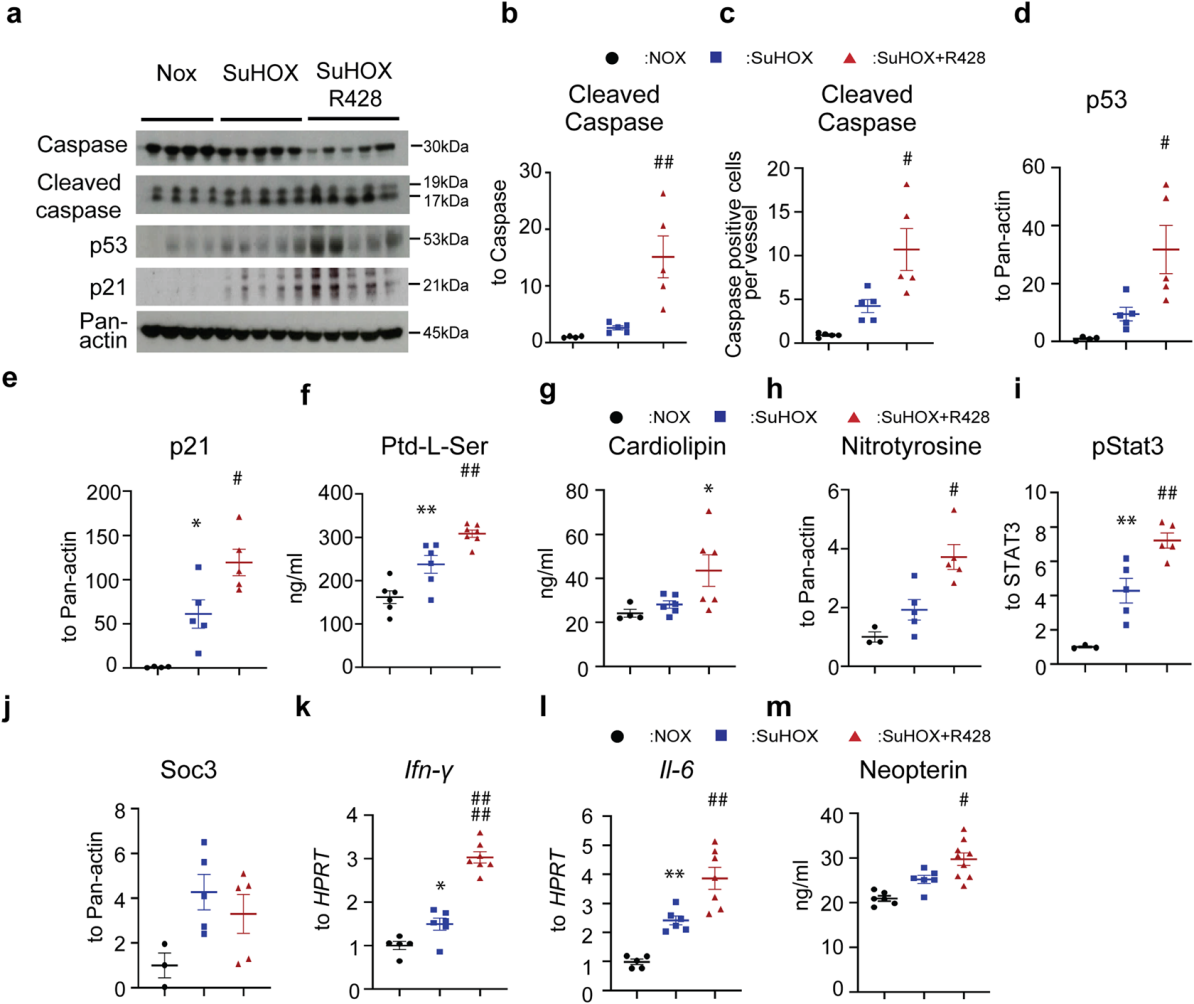


which replaces the previous incorrect version:
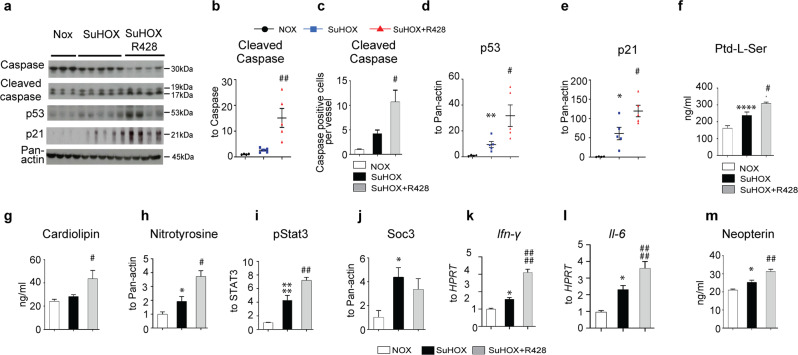


In addition, the upper and lower part of Fig. 5e contained different sample lane numbers and these western blots are now shown as separate panels.

The correct version of Fig. 5 is:
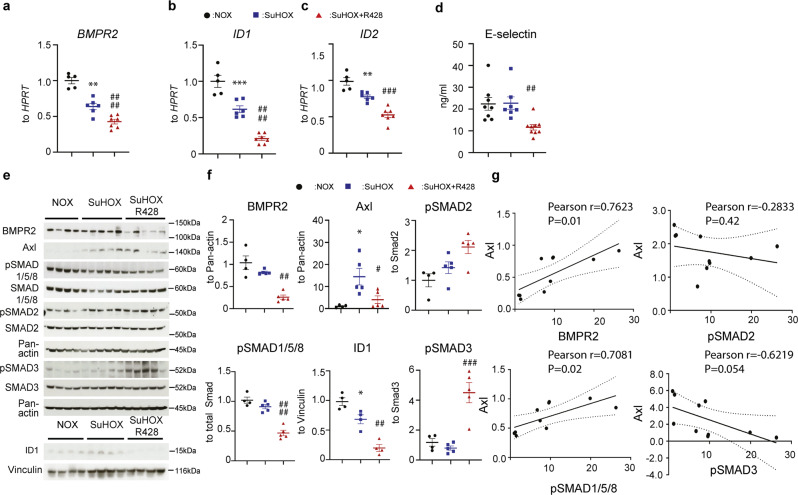


which replaces the previous incorrect version:
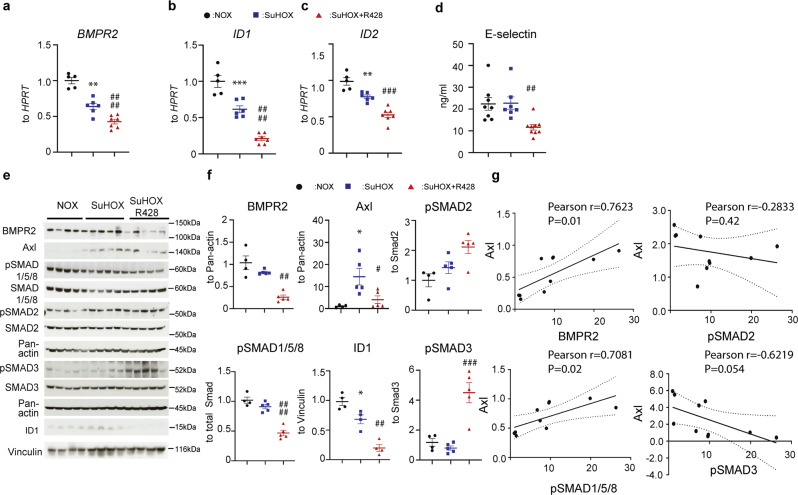


Both corrections do not affect the quantifications in the results section of the paper, the discussion of these results and the general message of the paper.

The figures have been corrected in both the PDF and HTML versions of the article.

