# Supplementary material for: Deficiency of Axl aggravates pulmonary arterial hypertension via BMPR2
Source: Commun Biol. 2021 Aug 24;4:1002. doi: 10.1038/s42003-021-02531-1 (PMC8385080; doi:10.1038/s42003-021-02531-1)
Supplement: Supplementary file 2 — Supplementary information. [file 42003_2021_2531_MOESM2_ESM.pdf]

Supplementary Information

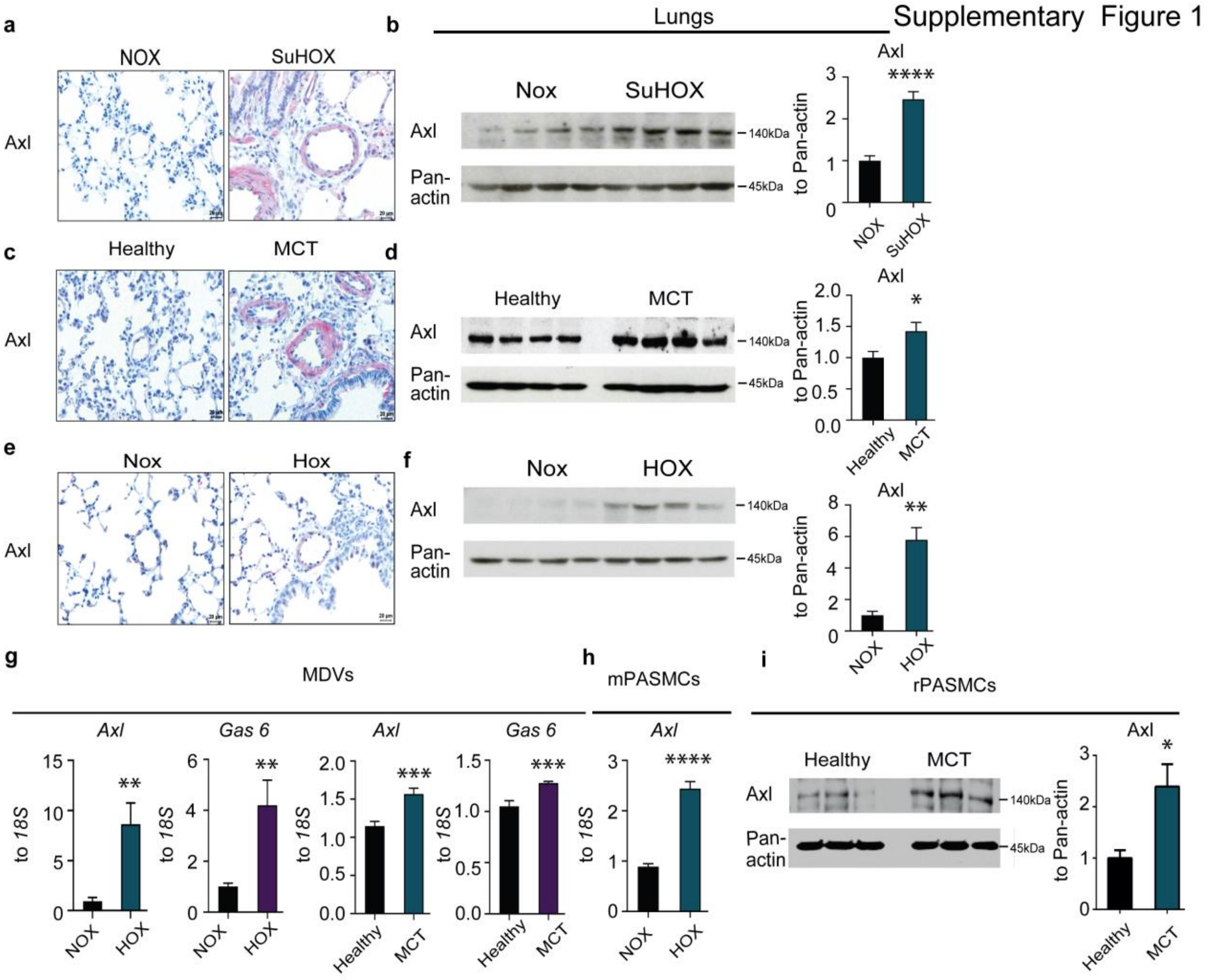

Supplementary Figure 1. Analyses of Axl expression in experimental pulmonary hypertension (PH). a, c, e Representative images of immunohistological staining of Axl expression in lung sections derived from **a** the Sugen 5416/hypoxia (SuHOX) experimental rat PAH model (normoxic rats [NOX], n = 3; SuHOX, n = 3), **c** the monocrotaline (MCT) experimental PAH rat model (healthy, n = 3; MCT, n = 3), and the chronic-hypoxia experimental PH mouse model (heathy, n = 3; MCT, n = 3). **b, d, f** Western blot analysis and subsequent densitometric quantification of Axl expression in lung homogenates from **b** the SuHOX (NOX, n = 8; SuHOX, n =9), **d** the MCT (healthy, n =8; MCT, n = 8), and the chronic-hypoxia (NOX, n = 4; HOX, n =4) experimental PH models. Pan-actin served as a loading control. Statistical analysis was performed using Student's t-test; ****P < 0.0001 for SuHOX versus NOX, *P <

0.05 for MCT versus healthy, and **P <0.01 for HOX versus NOX **g** Real-time quantitative PCR analyses of *Axl* mRNA expression from laser-assisted microdissected vessels (MDVs) in HOX (n = 6) versus NOX (n = 6) mouse lungs; and healthy control (n = 5) and MCT-injected rat lungs (n = 5). Expression levels were normalized to the *18S* house-keeping gene. **h** Relative mRNA expression of Axl from mouse pulmonary arterial smooth muscle cells (PASMCs), normalized to *18S,* as a reference gene. Data from three independent experiments are presented as the n-fold change ($2^{-\Delta\Delta Ct}$) compared with NOX control cells. **i** Western blot analysis and subsequent densitometric quantification of Axl expression in PASMCs isolated from the MCT rats (healthy, n = 6; MCT, n = 6). Pan-actin served as a loading control. Statistical analysis was performed using Student's t-test; ***P < 0.001, *P < 0.05 for MCT versus healthy controls and **P <0.01, ****P < 0.0001 for HOX versus NOX. All the data represent mean ± SEM.

Supplementary Figure 2

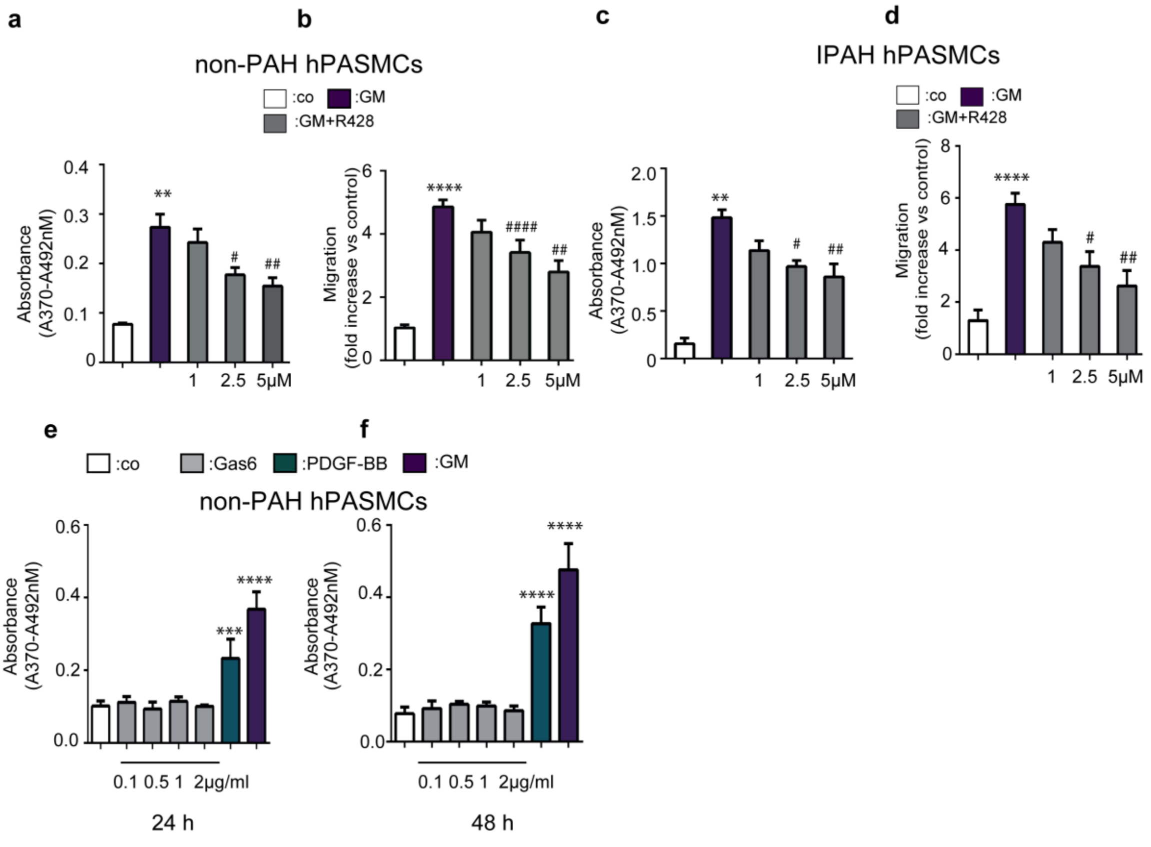

Supplementary Figure 2. Evaluation of the impact of R428 and growth arrest-specific protein 6 (Gas6) on proliferation and migration of human pulmonary arterial smooth muscle cells. Cultured human pulmonary arterial smooth muscle cells (hPASMCs) from **a, b** healthy individuals and **c, d** patients with idiopathic pulmonary arterial hypertension (IPAH) were exposed to growth medium (GM, consisting of 5% fetal bovine serum [FBS], human basic fibroblast growth factor [FGF-2], human epidermal growth factor [EGF], and insulin) for 24 h with or without R428 at indicated concentrations. Control cells were exposed to dimethyl sulfoxide (DMSO; co). **a, c** Proliferation potential was assessed by 5-bromo-2'-deoxyuridine (BrdU) colorimetric enzyme-linked immunosorbent assay (ELISA). Four hours after BrdU addition, assessment of DNA synthesis was completed [$A_{370nm} - A_{492nm}$]. Data from four independent experiments performed in triplicate are presented as mean ± SEM. **b, d** Migration efficiency was determined by transwell migration assays. Human PASMCs were exposed to GM for 16 h with or without increasing concentrations of R428, as indicated. Data from three independent experiments performed in triplicate are presented as mean ± SEM of the *n*-fold change normalized to DMSO-treated control cells. Statistical analysis was performed using one-way analysis of variance (ANOVA) with Newman–Keuls post-hoc test for multiple comparisons. $^{**}P < 0.01$ and $^{****}P < 0.0001$ versus DMSO-treated control cells; $^{\#}P < 0.05$, $^{\#\#}P < 0.01$ and $^{\#\#\#\#}P < 0.0001$ versus GM-treated cells. **e, f** Cultured hPASMCs from healthy individuals were stimulated for 24 h and 48 h with indicated concentrations of Gas6, PDGF-BB (30 ng/ml), and GM with or without R428 at indicated concentrations. Control cells were left in serum-free conditions (co). Proliferation potential was assessed by BrdU colorimetric ELISA. Four hours after BrdU addition, assessment of DNA synthesis was completed [$A_{370nm} - A_{492nm}$]. Data from three independent experiments performed in triplicate are presented as mean ± SEM. Statistical analysis was performed using one-way ANOVA with Newman–Keuls post-hoc test for multiple comparisons. $^{***}P < 0.001$ and $^{****}P < 0.0001$. All the data represent the mean ± SEM.

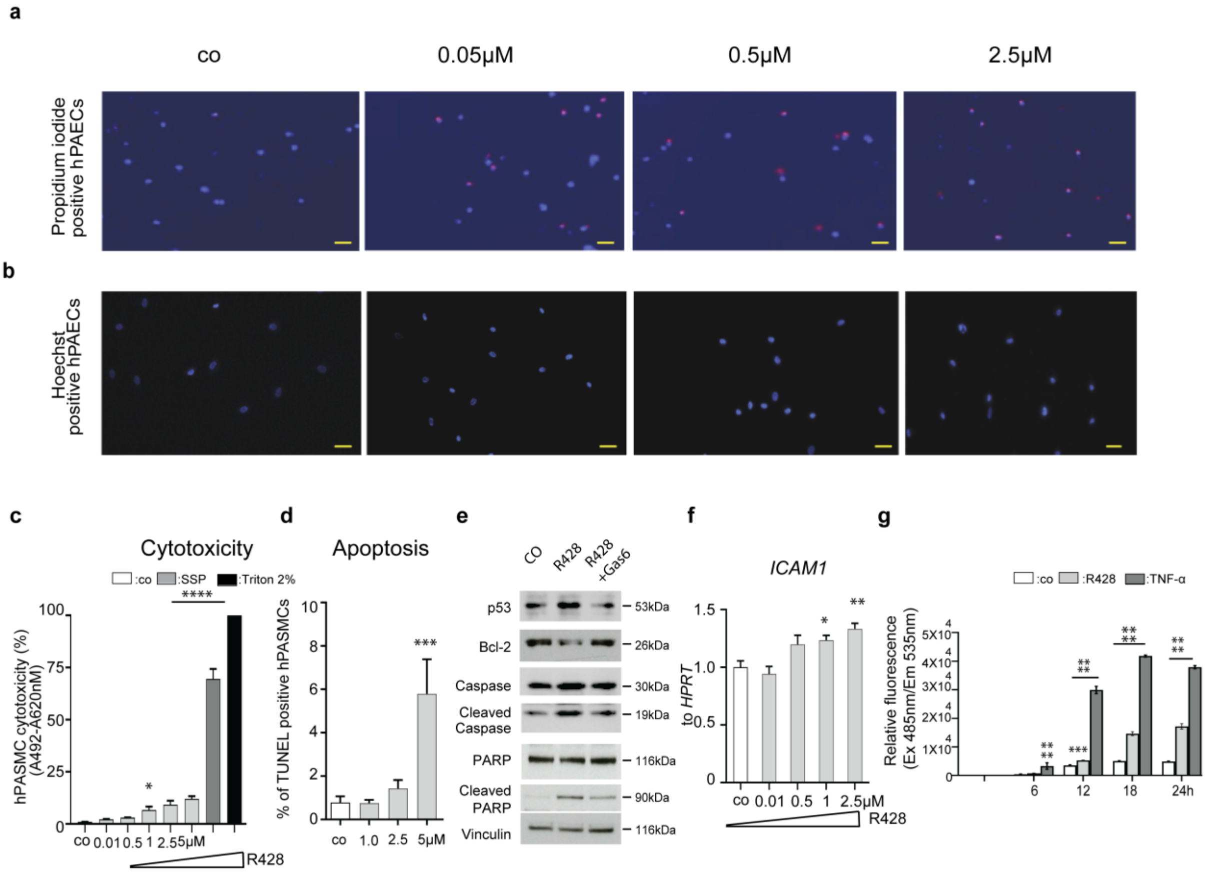

Supplementary Figure 3. Evaluation of the impact of R428 and growth arrest-specific protein 6 (Gas6) on cytotoxicity, apoptosis and permeability of human pulmonary vascular cells. Representative images of immunofluorescent staining with (**a**) propidium iodide (red) and (**b**) Hoechst (blue) of human pulmonary arterial smooth muscle cells (hPAECs) exposed to increasing concentrations of R428. Control cells were exposed to dimethyl sulfoxide (DMSO; co). DNA in (**a**) was visualized using 4',6'-diamidino-2-phenylindole (DAPI; blue). Scale bar: 50 µm. **c** Cytotoxicity in human pulmonary arterial smooth muscle cells (hPASMCs) was determined by detecting LDH release into the cell culture media [$A_{492nm} – A_{620nm}$]. Cells treated with Staurosporine (SSP) were used as positive control. Data are reported as percentage release of LDH compared to DMSO-treated control (co) cells. Data from three independent experiments performed in triplicates are presented. Statistical analysis was performed using one-way ANOVA with Newman–Keuls post-hoc test for multiple comparisons; *$P < 0.05$, ****$P < 0.0001$. **d** Quantitative analyses of TUNEL-positive hPASMCs

exposed to increasing concentrations of R428. Data from three independent experiments performed in triplicate are presented as mean ± SEM of the *n*-fold change normalized to DMSO-treated control (co) cells. Statistical analysis was performed using one-way analysis of variance (ANOVA) with Newman–Keuls post-hoc test for multiple comparisons. ***$P < 0.001$ versus DMSO-treated control cells. **e** Representative images of western blots of apoptotic markers p53, B-cell lymphoma 2 (Bcl-2), Caspase, Cleaved caspase, and poly (ADP-ribose) polymerase (PARP) expression in cells exposed to R428 (5 µM) for 24 h and subsequently stimulated with growth arrest-specific 6 (Gas6; 200 ng/ml). Vinculin served as a loading control. **f** Relative mRNA expression of intercellular adhesion molecule 1 (*ICAM1*) expression in cultured healthy hPASMCs treated for 24 h with indicated concentrations of R428. Data are presented as mean ± SEM of the *n*-fold change ($2^{-\Delta\Delta Ct}$) normalized to hypoxanthine guanine phosphoribosyl transferase (*HPRT*) as a reference gene, compared with DMSO-treated control cells. Data from three independent experiments are presented. *$P < 0.05$ and **$P < 0.01$ versus DMSO treated control cells. Statistical analysis was performed using one-way ANOVA with Newman–Keuls post-hoc test for multiple comparisons. **g** Quantitative analyses of vascular permeability determined by FITC-dextran permeability assay. Data from two independent experiments performed in fourplicate are presented. ***$P < 0.001$ and ****$P < 0.0001$ versus DMSO-treated control cells. For statistical analysis two-way analysis of variance with Tukey`s post-hoc test for multiple comparisons was performed. All the data represent the mean ± SEM.

Supplementary Figure 4

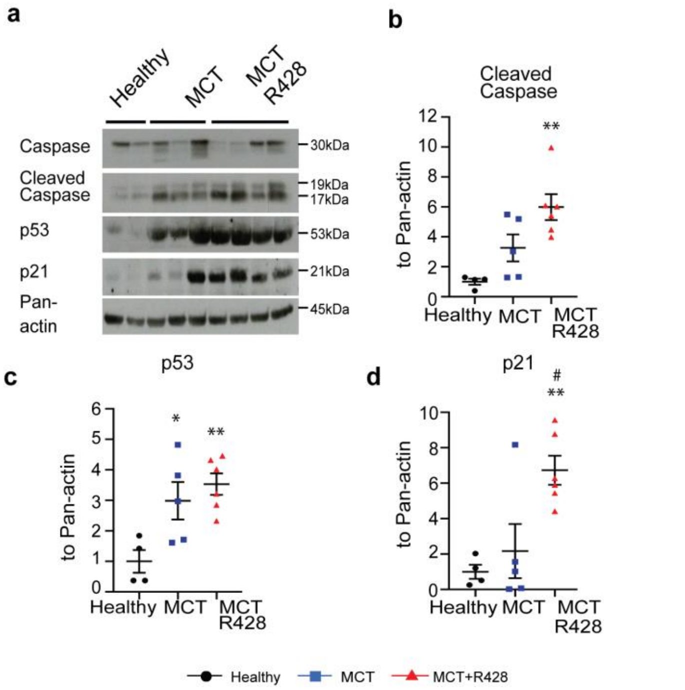

Supplementary Figure 4. Effect of R428 on apoptotic response in experimental Monocrotaline rat model of pulmonary hypertension (PH). a Representative western blots of markers of apoptosis in total lung homogenates of rats exposed to monocrotaline (MCT) injection and controls (healthy, *n* = 4; MCT, *n* = 5; MCT + R428, *n* = 6). **b** Subsequent densitometric quantification of Cleaved Caspase, **c** p53 and **d** p21 expression in total lung homogenates of rats exposed to MCT injection and controls (healthy, *n* = 4; MCT, *n* = 5; MCT+R428, *n* = 6). Data are presented as mean ± SEM and statistical analysis was performed using one-way analysis of variance with Tukey's post-hoc test for multiple comparisons. *$P < 0.05$ for MCT versus healthy controls; **$P < 0.01$ for MCT + R428 versus healthy controls; $^{\#}P < 0.05$ for MCT + R428 versus MCT. Pan-actin served as a loading control. All the data represent the mean ± SEM.

Supplementary Figure 5

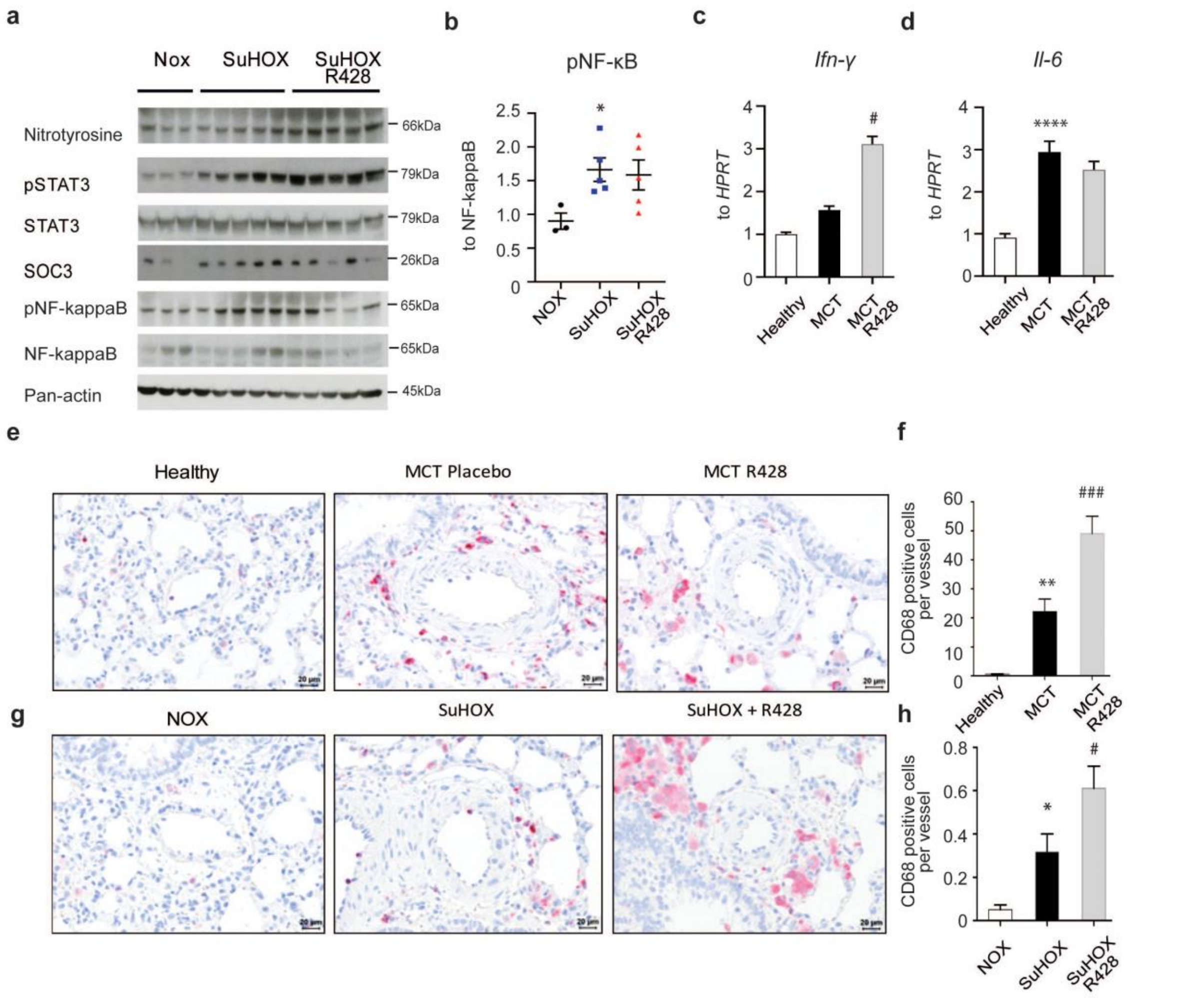

Supplementary Figure 5. Effect of R428 on pro-inflammatory response in experimental rat models of pulmonary hypertension (PH).

a Representative western blots of Nitrotyrosine, phosphorylated signal transducer and activator of transcription 3 (phospho-STAT3), suppressor of cytokine signaling 3 (SOCS3), and phospho- and total p65 of nuclear factor kappa-light-chain-enhancer of activated B-cells pathway, and **b** subsequent densitometric quantification of phospho-65 in total lung homogenates of all three experimental groups of the Sugen 5416/hypoxia (SuHOX) experimental rat model of PH (healthy, $n = 3$, SuHOX, $n = 5$, SuHOX + R428, $n = 5$). The levels of phospho-STAT3 were controlled by total STAT3 protein. Pan-actin served as a loading control. **c, d** Real-time quantitative PCR analyses of **c** interferon γ (*Ifn-γ)* and **d**

interleukin 6 (*Il-6*) expression in mRNA from lungs of rats exposed to monocrotaline (MCT) and controls. Data are presented as mean ± SEM of the *n*-fold change ($2^{-\Delta\Delta Ct}$) normalized to hypoxanthine guanine phosphoribosyl transferase (*HPRT*) as a reference gene, compared with healthy controls (healthy, *n* = 5; MCT, *n* = 5; MCT + R428, *n* = 6). **e**, **g** Representative images and **f, h** quantitative analyses of of immunohistological staining of CD68 expression in lung sections derived from the MCT and the SuHOX experimental rat models of PH upon R428 treatment ([**e,f**] healthy, *n* = 6; MCT, *n* = 5; MCT+R428, *n* = 6; and [**g,h**] NOX, *n* = 6; SuHOX, *n* = 6; SuHOX+R428, *n* = 6). Statistical analysis was performed using one-way analysis of variance with Tukey's post-hoc test for multiple comparisons. **P* < 0.05, ***P* < 0.01, and *****P* < 0.0001 for MCT and SuHOX versus healthy and normoxic (NOX) controls, respectively; #*P* < 0.05 and ###*P* < 0.001 versus MCT + Placebo (MCT) and SuHOX controls, respectively. All the data represent the mean ± SEM.

Supplementary Figure 6

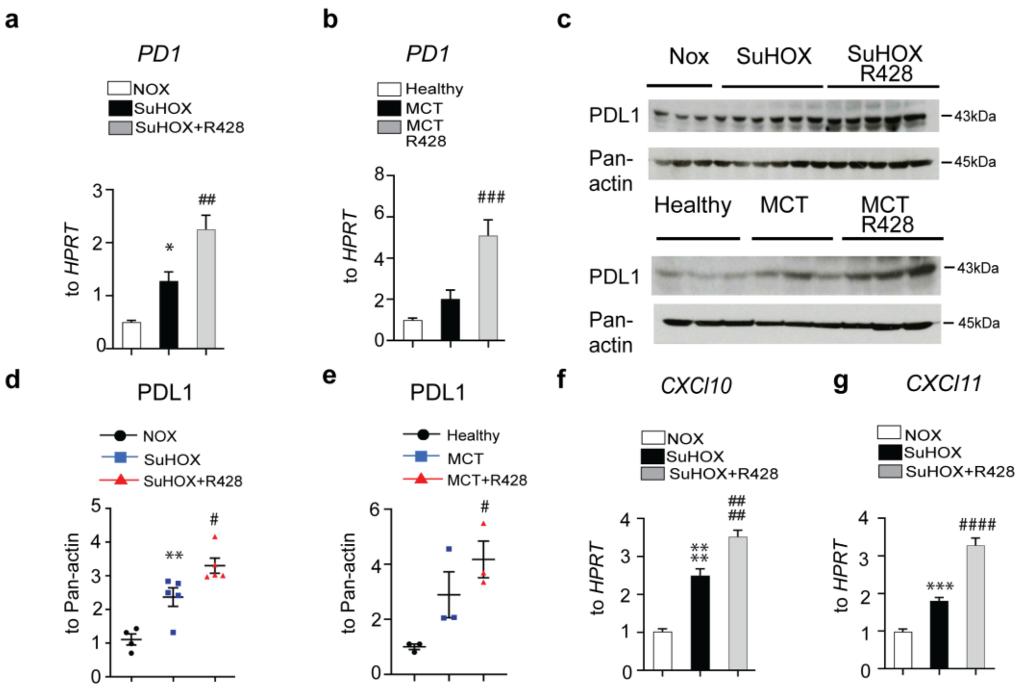

Supplementary Figure 6. Effect of R428 on programmed cell death ligand 1 (PDL1)/programmed cell death 1 (PD1) axis in experimental rat models of pulmonary hypertension (PH). Real-time quantitative PCR analyses of mRNA expression of *Pd1* in total lung homogenates of **a** Sugen 5416/hypoxia (SuHOX) and **b** monocrotaline (MCT) experimental rat models of PH. Data are presented as mean ± SEM of the *n*-fold change ($2^{-\Delta\Delta Ct}$) normalized to hypoxanthine guanine phosphoribosyl transferase (*HPRT*) as a reference gene, compared with normoxic (NOX, *n* = 5; SuHOX, *n* = 6; SuHOX + R428, *n* = 7) or healthy control rats (healthy, *n* = *6*; MCT, *n* = 5; MCT+R428, *n* = 6). **c** Western blots and **d, e** subsequent densitometric quantification of PDL1 in total lung homogenates of **d** SuHOX and **e** MCT experimental rat models upon R428 administration. Representative samples from all three experimental groups are shown (NOX, *n* = 4; SuHOX, *n* = 5; SuHOX + R428, *n* = 5 and healthy, *n* = 3; MCT, *n* = 3; MCT+R428, *n* = 3). Pan-actin served as a loading control. **f, g** Real-time quantitative PCR analyses of mRNA expression of C-X-C motif chemokine ligands *Cxcl10* and *Cxcl11* from total lung homogenates of SuHOX experimental rat model of pulmonary hypertension. Data are presented as mean ± SEM of the *n*-fold change ($2^{-\Delta\Delta Ct}$) normalized to *HPRT* as a reference gene, compared with normoxic controls (NOX, *n* = 6; SuHOX, *n* = 6; SuHOX + R428, *n* = 7). Statistical analysis was performed using one-way analysis of variance with Tukey's post-hoc test for multiple comparisons. *$P < 0.05$, **$P < 0.01$, ***$P < 0.001$, and ****$P < 0.0001$ for SuHOX versus NOX controls; $^{\#}P < 0.05$, $^{\#\#\#}P < 0.001$ for MCT + R428 versus MCT controls; $^{\#}P < 0.05$, $^{\#\#}P < 0.01$, and $^{\#\#\#\#}P < 0.001$ for SuHOX +R428 versus SuHOX controls. All the data represent the mean ± SEM.

Supplementary Figure 7

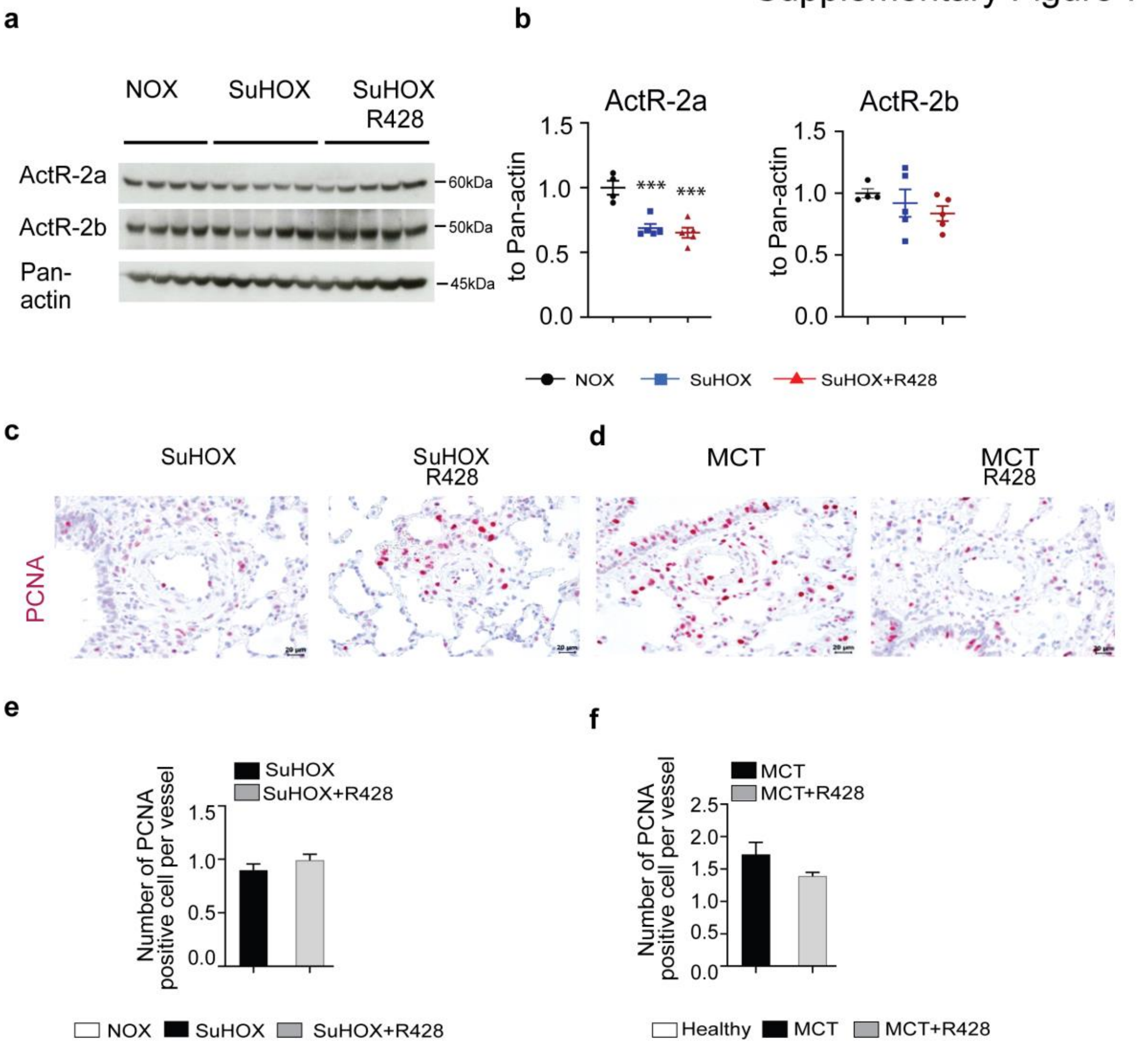

Supplementary Figure 7. Analyses of activin A receptor type 2A (ActR-2a) and activin A receptor type 2B (ActR-2b) expression in human pulmonary arterial smooth muscle cells (hPASMCs). a Western blots and **b** subsequent densitometric quantification of ActR-2a and ActR-2b in total lung homogenates of Sugen 5416/hypoxia (SuHOX) experimental rat model upon R428 administration. Representative samples from all three experimental groups are shown (NOX, $n = 4$; SuHOX + placebo [SuHOX], $n = 5$; SuHOX + R428, $n = 5$). Pan-actin served as a loading control. Statistical analysis was performed using one-way analysis of variance with Tukey's post-hoc test for multiple comparisons. ***$P < 0.001$ for SuHOX (or SuHOX+R428) versus NOX **c,d** Representative images of immunohistological staining of proliferating cell nuclear antigen (PCNA) in lung sections derived from **h** the SuHOX (SuHOX, $n = 6$; SuHOX+R428, $n = 6$) and **i** the MCT (MCT,

n = 5; MCT+R428, *n* = 6) experimental rat PH models. Scale bars: 20µm. **e, f** Quantitative analysis of immunofluorescent staining of pulmonary vascular proliferative cells using PCNA antibody. Statistical analysis was performed using Student's t-test. All the data represent the mean ± SEM.

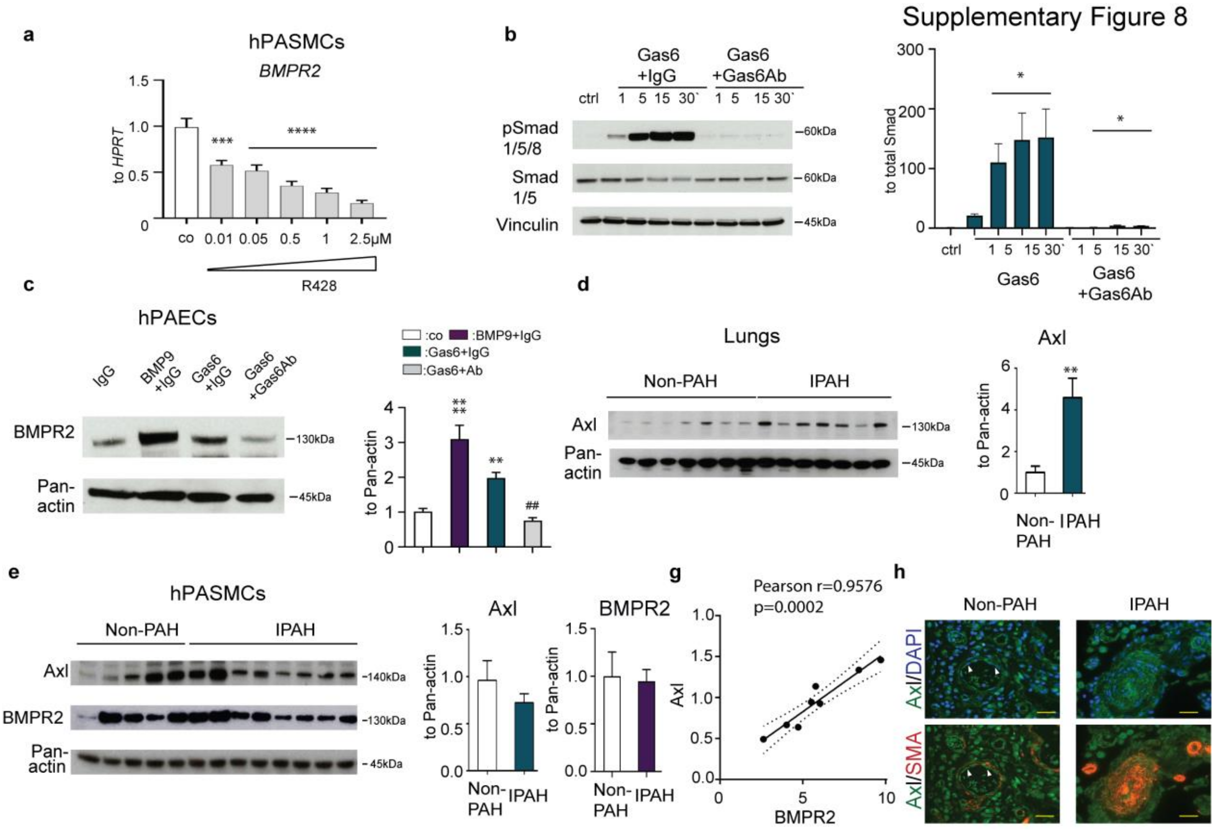

Supplementary Figure 8. Analyses of *BMPR2* and Axl expression in human pulmonary arterial smooth muscle cells (hPASMCs). a Real-time quantitative PCR analyses of mRNA expression of bone morphogenetic protein receptor 2 (*BMPR2*) in healthy cultured hPASMCs treated for 24 h with indicated concentrations of R428. Data are presented as mean ± SEM of the *n*-fold change ($2^{-\Delta\Delta Ct}$) normalized to hypoxanthine guanine phosphoribosyl transferase (*HPRT*) as a reference gene, compared with DMSO-treated control cells. Data from three independent experiments are presented. Statistical analysis was performed using one-way ANOVA with Newman–Keuls post-hoc test for multiple comparisons. ****P* < 0.001 and *****P* < 0.0001 versus DMSO treated control (co) cells. **b** Western blot analysis and subsequent

densitometric quantification of Smad1/5/8 activation in hPAECs stimulated with Gas6 and pre-treated with Gas6Ab and IgG control. Data from three independent experiments are presented. **c** Western blot analysis and subsequent densitometric quantification of BMPR2 expression in hPAECs stimulated for 48h with BMP9 and Gas6 followed by pre-treatment with Gas6Ab and IgG control. Data from three independent experiments are presented. **b, c** Statistical analysis was performed using one-way ANOVA with Newman–Keuls post-hoc test for multiple comparisons. $^{*}P < 0.05$ and $^{****}P < 0.0001$ versus control (co) IgG treated cells; $^{\#\#}P < 0.01$ versus Gas6+Gas6Ab treated cells. **d** Western blot analysis and subsequent densitometric quantification of Axl receptor expression from total lung homogenates isolated from patients with idiopathic pulmonary arterial hypertension (IPAH; $n = 8$) and non-PAH control individuals ($n = 7$). Statistical analysis was performed using Student's t-test; $^{**}P < 0.01$. **e** Western blot analysis and subsequent densitometric quantification of Axl and BMPR2 receptor expression in hPASMCs of patients with idiopathic pulmonary arterial hypertension (IPAH; $n = 8$) and non-PAH control individuals ($n = 5$). Pan-actin served as a loading control. **g** Correlation analysis of Axl and BMPR2 expression in hPASMCs. Pearson correlation coefficient (r value) and the significance (P value) of correlation analysis is indicated on the graph. **h** Representative examples of immunofluorescence staining of Axl (green) expression in tissue sections of non-PAH (n = 4) and IPAH (n = 4) human lung specimens. Sections were double stained with α-smooth muscle actin (α-SMA) (red) antibody. Nuclei were visualized using 4',6'-diamidino-2-phenylindole (DAPI; blue). Scale bars: 50µm. All the data represent the mean ± SEM.

Gene	Host	Company	Cat. No.	Dilution in 5% BSA/TBS/T
Actr2A	Ms	Santa Cruz Biotechnology, Dallas, US	sc-515826	1:500
Actr2B	Ms	Santa Cruz Biotechnology, Dallas, US	sc-134245	1:500
AXL (C89E7)	Rb	Cell Signaling Technology, Inc., Danvers, US	#8661S	1:1000
BCL-2 (C-2)	Ms	Santa Cruz Biotechnology, Dallas, US	sc-7382	1:500
BCL-2 (N-19)	Rb	Santa Cruz Biotechnology, Dallas, US	sc-492	1:500
BMPR2	Rb	Abbexa Ltd, Cambridge, UK	abx007889	1:1000
BMPR2 (E-1)	Ms	Santa Cruz Biotechnology, Dallas, US	sc-393304	1:1000
BMPR2	Ms	BD Biosciences, San Jose, CA, US	612292	1:500
Caspase-3	Rb	Cell Signaling Technology, Inc., Danvers, US	#9662S	1:1000
Cleaved Caspase-3 (Asp175)	Rb	Cell Signaling Technology, Inc., Danvers, US	#9661S	1:500
Cyclin D1 (H-295)	Rb	Santa Cruz Biotechnology, Dallas, US	sc-753	1:500
Gas6	Gt	R&D Systems, Minneapolis, US	AF885	1:500
ID1 (B-8)	Ms	Santa Cruz Biotechnology, Dallas, US	sc-133104	1:500

NFκB p65 (F-6)	Ms	Santa Cruz Biotechnology, Dallas, US	sc-8008	1:500
Nitrotyrosine (1A6)	Rb	Merck Millipore, Burlington, MA, US	06-284	1:500
p21 (F-5)	Ms	Santa Cruz Biotechnology, Dallas, US	sc-6246	1:500
p27 (F-8)	Rb	Santa Cruz Biotechnology, Dalls, US	sc-1641	1:500
p53 (1C12)	Ms	Cell Signaling Technology, Inc., Danvers, US	#2524S	1:500
Pan-Actin	Rb	Cell Signaling Technology, Inc., Danvers, US	#4968S	1:2000
PD-L1 (CD274)	Rb	Abbexa Ltd, Cambridge, UK	abx001385	1:1000
pAXL (Y779)	Ms	R&D Systems, Minneapolis, US	MAB6965	1:500
pSmad 1/5/9	Rb	Cell Signaling Technology, Inc., Danvers, US	#13820S	1:500
pSmad 2	Rb	Cell Signaling Technology, Inc., Danvers, US	#3108S	1:500
pSmad 3	Rb	Cell Signaling Technology, Inc., Danvers, US	#9520S	1:500
pSTAT3 (S727)	Rb	Cell Signaling Technology, Inc., Danvers, US	#9134S	1:500
pNFκB p65 (S536)	Ms	Santa Cruz Biotechnology, Dallas, US	sc-136548	1:500
Smad 1/5/8 (N-18)	Rb	Santa Cruz Biotechnology, Dallas, US	sc-6031-R	1:1000

Smad2/3	Rb	Cell Signaling Technology, Inc., Danvers, US	#3102	1:1000
SOCS3	Rb	Cell Signaling Technology, Inc., Danvers, US	#2923	1:500
STAT3 (79D7)	Rb	Cell Signaling Technology, Inc., Danvers, US	#4904S	1:1000

Supplementary Table 1: List with primary antibodies used for Western blot analyses.

**Gene**	**Species**	**Forward**	**Reverse**
18S	*Homo sapiens*	CTCAACACGGGAAACCTCAC	CGCTCCACCAAACTAAGAACG
AXL	*Homo sapiens*	GCAGGCTGAAGAAAGTCCCT	CCTGTTCATCCTCACCCAGG
AXL	*Rattus norvegicus*	TCCACCATCTGGTGAGTGA	CCTCCCCATATCTCGTCTCCT
BMP R2	*Homo sapiens*	CCACTGAATCGCTGGACTGT	ATGGTTGTAGCAGTGCCTCC
BMP R2	*Rattus norvegicus*	CACTACGGCTGCTTCCCAGAA	AGACCAGCACCCTTGTTTCAC
B2M	*Homo sapiens*	CTCGCGCTACTCTCTCTTTCT	CATTCTCTGCTGGATGACGTG
Cxcl10	*Rattus norvegicus*	TGCAAGTCTATCCTGTCCGC	TGACCTTCTTTGGCTCACCG

Cxcl11	*Rattus norvegicus*	CCGAGTAACGGCTGTGACAA	TATGAGGCGAGCTTGCTTGG
E-Sel	*Homo sapiens*	TGGTGAGGTGTGCTCATTCC	AGAGAAATGGCAGGTGGAGC
GAPDH	*Homo sapiens*	CAGAAGACTGTGGATGGCCC	AGTGTAGCCCAGGATGCCCT
GAS6	*Rattus norvegicus*	CCCCCGTGATTAGACTACGC	GATCCAGGTGCTATCCGAGC
HPRT	*Homo sapiens*	TGAGGATTTGGAAAGGGTGTT	ATGTAATCCAGCAGGTCAGCA
HPRT	*Rattus norvegicus*	AGGCCAGACTTTGTTGGATT	GCTTTTCCACTTTCGCTGAT
ICAM1	*Homo sapiens*	GGTAGCAGCCGCAGTCATAA	TCCCTTTTTGGGCCTGTTGT
ID1	*Rattus norvegicus*	CAGCTGGAGCTGAACTCTGA	GCTCCTTGAGGCGTGAGTAG
ID1	*Homo sapiens*	CGGAATCCGAAGTTGGAACC	CAGCGACACAAGATGCGAT
ID2	*Rattus norvegicus*	CCAGAGACCTGGACAGAACC	CGACATAAGCTCAGAAGGGAAT
ID2	*Homo sapiens*	TGAAAGCCTTCAGTCCCGTG	TGAGCTTGGAGTAGCAGTCG
***IFN*γ**	*Rattus norvegicus*	GTGTCATCGAATCGCACCTG	ACCGACTCCTTTTCCGCTTC
IL-6	*Rattus norvegicus*	AGCGATGATGCACTGTCAGA	GGAACTCCAGAAGACCAGAGC
PD-1	*Rattus*	CCCAACGGACATGACTTCCA	ATGGCCCCACAGAGGTAGAT

	norvegicus		

Supplementary Table 2: List with primer sequences used for quantitative real-time PCR analyses.

Product	Company	Cat. No.
PathScan Phospho-Axl (panTyr) Sandwich ELISA	Cell Signaling Technology, MA, USA	7042C
In Situ Cell Death Detection Kit TMR red	Roche, Basel, Switzerland	12156792910
Rat Anti-cardiolipin antibody IgG (ACA-IgG) ELISA	MyBioSource, San Diego, CA, USA	MBS265605
Rat Neopterin ELISA	MyBioSource, San Diego, CA, USA	MBS2515927
Rat Phosphatidylserine ELISA	MyBioSource, San Diego, CA, USA	MBS024773
Recombinant Human/Murine/Rat BMP-2	PeproTech, London, UK	120-40
Recombinant Human BMP-4	PeproTech, London, UK	120-05
Recombinant Human BMP-6	PeproTech, London, UK	120-06
Recombinant Human BMP-7	PeproTech, London, UK	120-03
Recombinant Human BMP-9	Biolegend, San Diego, CA, USA	553102
Recombinant Human BMP-10	R&D Systems, Minneapolis, USA	885-GSB
Recombinant Human Gas6	PeproTech, London, UK	100-14
Recombinant Human PDGF-BB	PeproTech, London, UK	120-02

Supplementary Table 3: List with recombinant proteins and ELISA kits.
