# Supplementary material for: Deficiency of Axl aggravates pulmonary arterial hypertension via BMPR2
Source: Commun Biol. 2021 Aug 24;4:1002. doi: 10.1038/s42003-021-02531-1 (PMC8385080; doi:10.1038/s42003-021-02531-1)
Supplement: Supplementary file 3 — Description of Supplementary Files. [file 42003_2021_2531_MOESM3_ESM.pdf]

Description of Additional Supplementary Files

File name: Supplementary Data 1

Description: Data underlying the graphs of all experiments presented in the main Figures and Supplementary Figures.

File name: Supplementary Data 2

Description: All original WB images.
