# Supplementary material for: Deficiency of Axl aggravates pulmonary arterial hypertension via BMPR2
Source: Commun Biol. 2021 Aug 24;4:1002. doi: 10.1038/s42003-021-02531-1 (PMC8385080; doi:10.1038/s42003-021-02531-1)

Figure 1

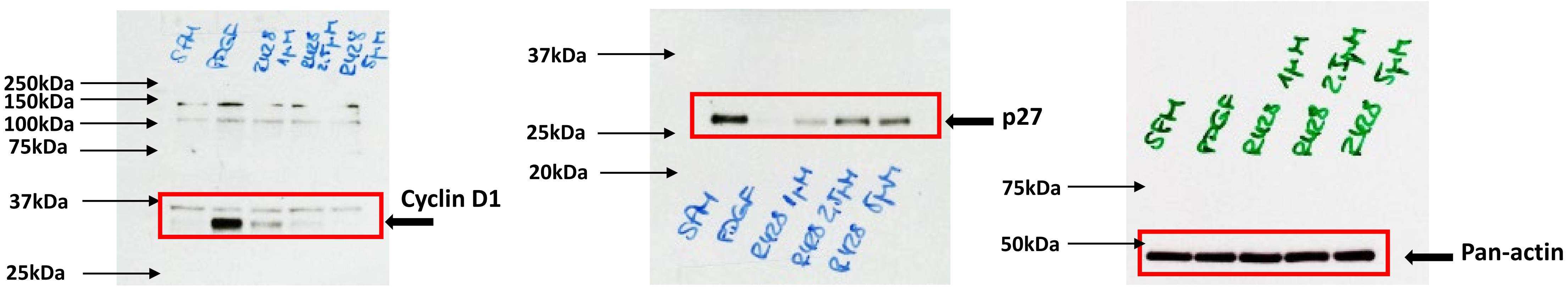
250kDa
150kDa
100kDa
75kDa
37kDa
25kDa
Cyclin D1
37kDa
25kDa
20kDa
p27
75kDa
50kDa
Pan-actin

Figure 4

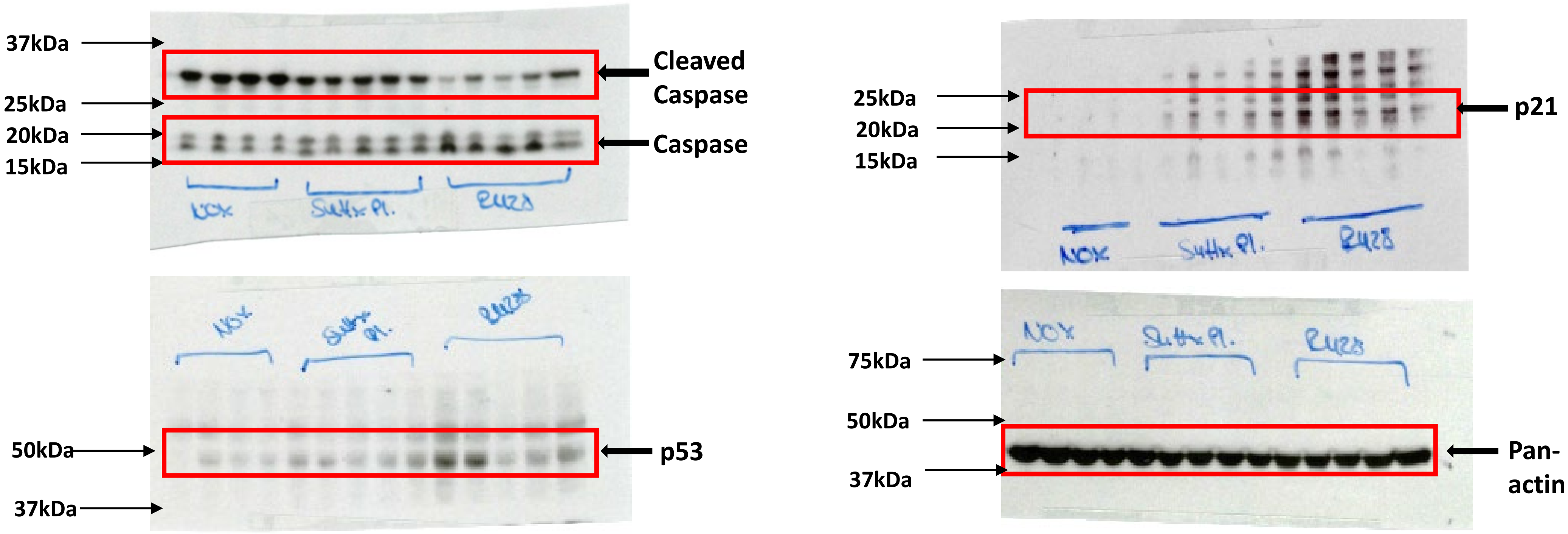

37kDa
25kDa
20kDa
15kDa
Cleaved Caspase
Caspase
50kDa
37kDa
p53
25kDa
20kDa
15kDa
p21
75kDa
50kDa
37kDa
Pan-actin

Figure 5

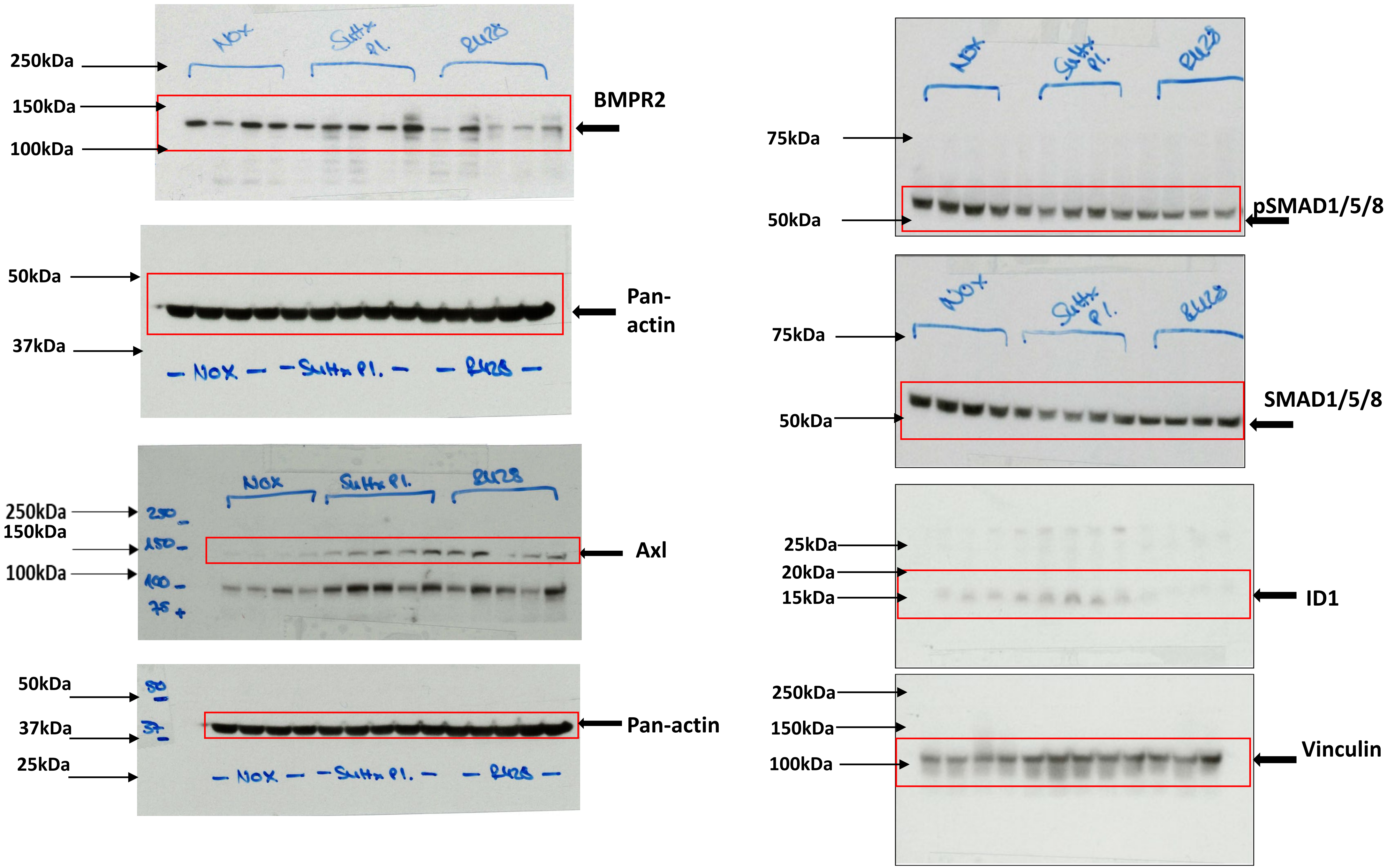

250kDa
150kDa
100kDa
BMPR2
50kDa
37kDa
Pan-actin
250kDa
150kDa
100kDa
Axl
50kDa
37kDa
25kDa
Pan-actin
75kDa
50kDa
pSMAD1/5/8
75kDa
50kDa
SMAD1/5/8
25kDa
20kDa
15kDa
ID1
250kDa
150kDa
100kDa
Vinculin

Figure 5

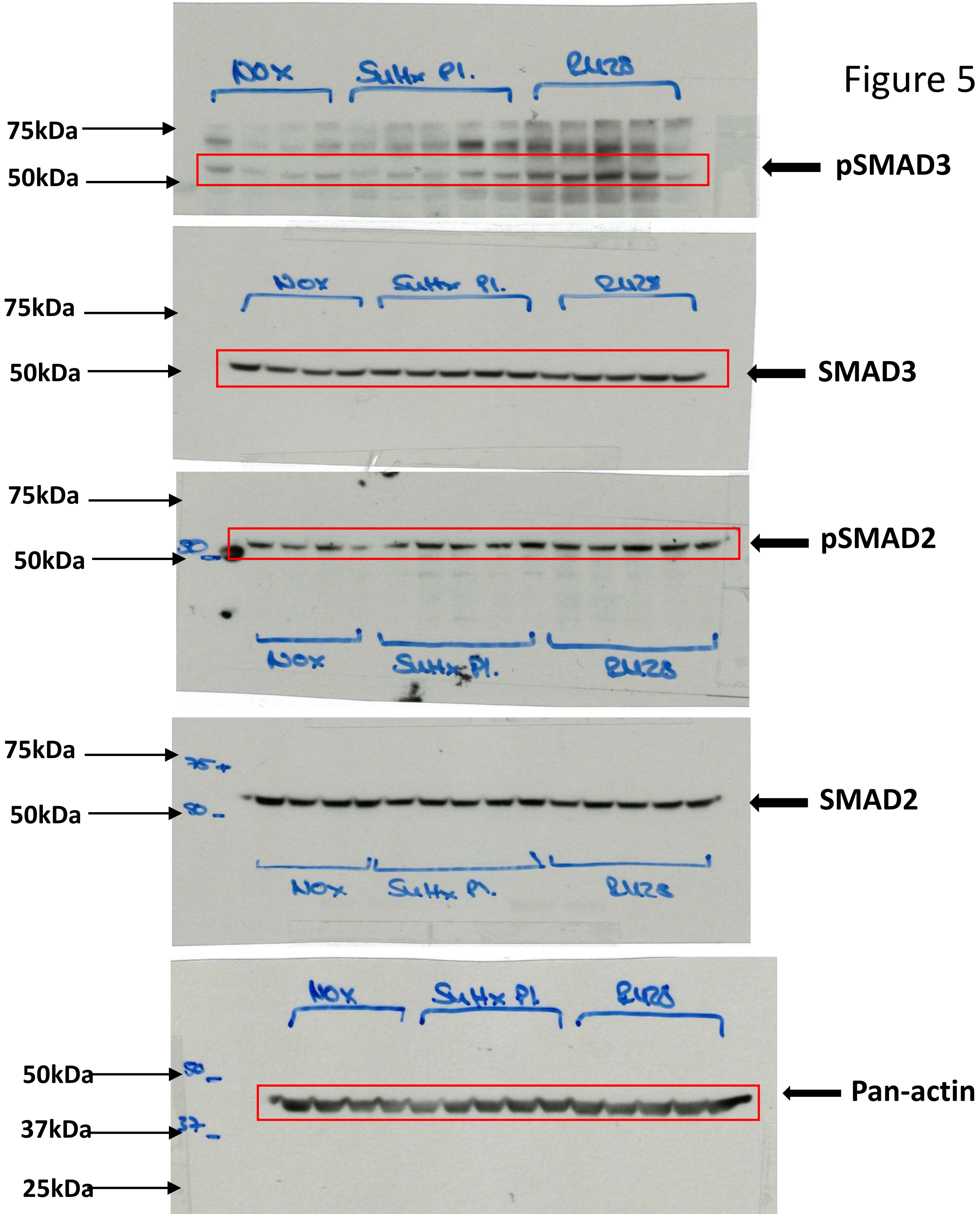
75kDa
50kDa
pSMAD3
75kDa
50kDa
SMAD3
75kDa
50kDa
pSMAD2
75kDa
50kDa
SMAD2
50kDa
37kDa
25kDa
Pan-actin

Figure 7

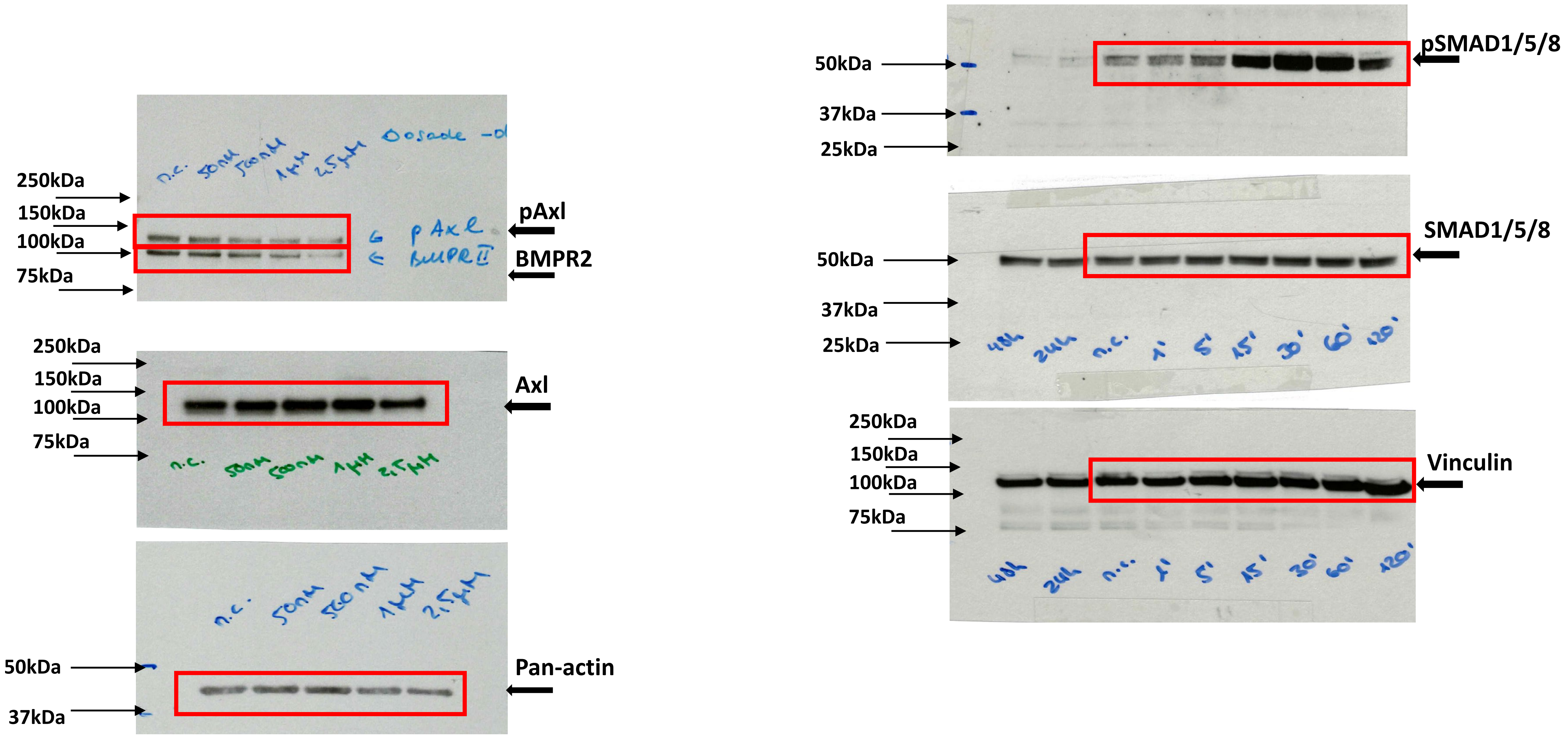
250kDa
150kDa
100kDa
75kDa
pAxl
BMPR2
250kDa
150kDa
100kDa
75kDa
Axl
50kDa
37kDa
Pan-actin
50kDa
37kDa
25kDa
pSMAD1/5/8
50kDa
37kDa
25kDa
SMAD1/5/8
250kDa
150kDa
100kDa
75kDa
Vinculin

Figure 7

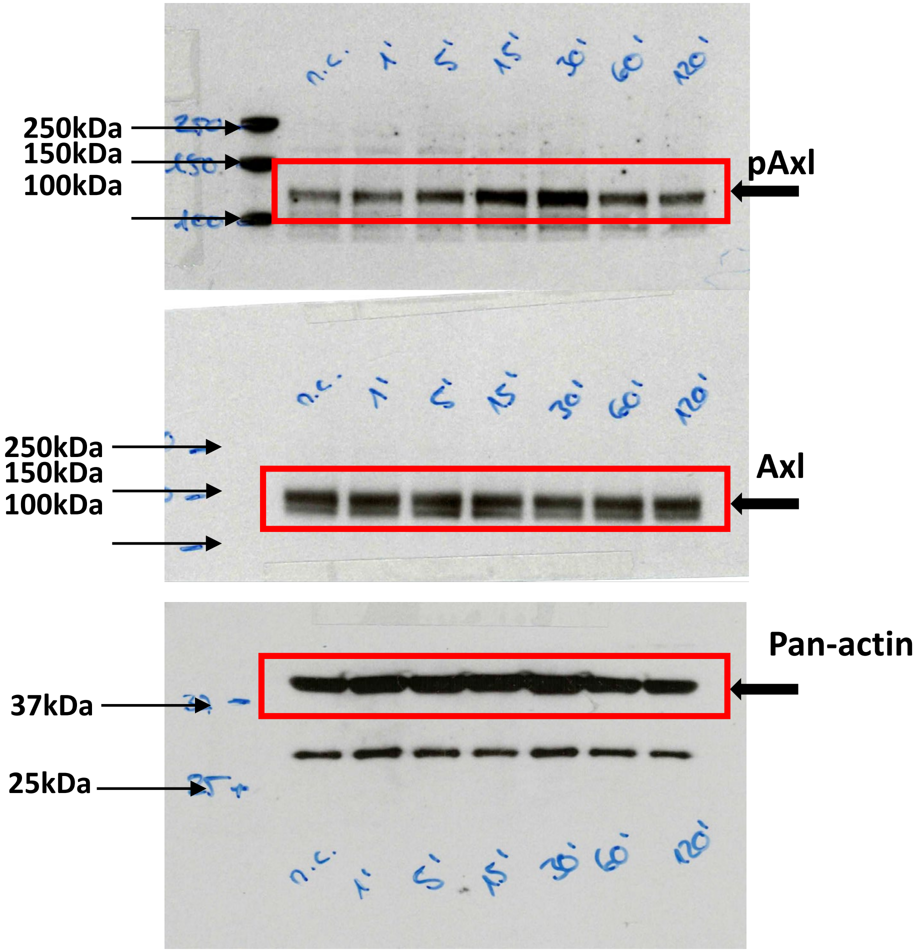
250kDa
150kDa
100kDa
pAxl
250kDa
150kDa
100kDa
Axl
Pan-actin
37kDa
25kDa

Figure 7

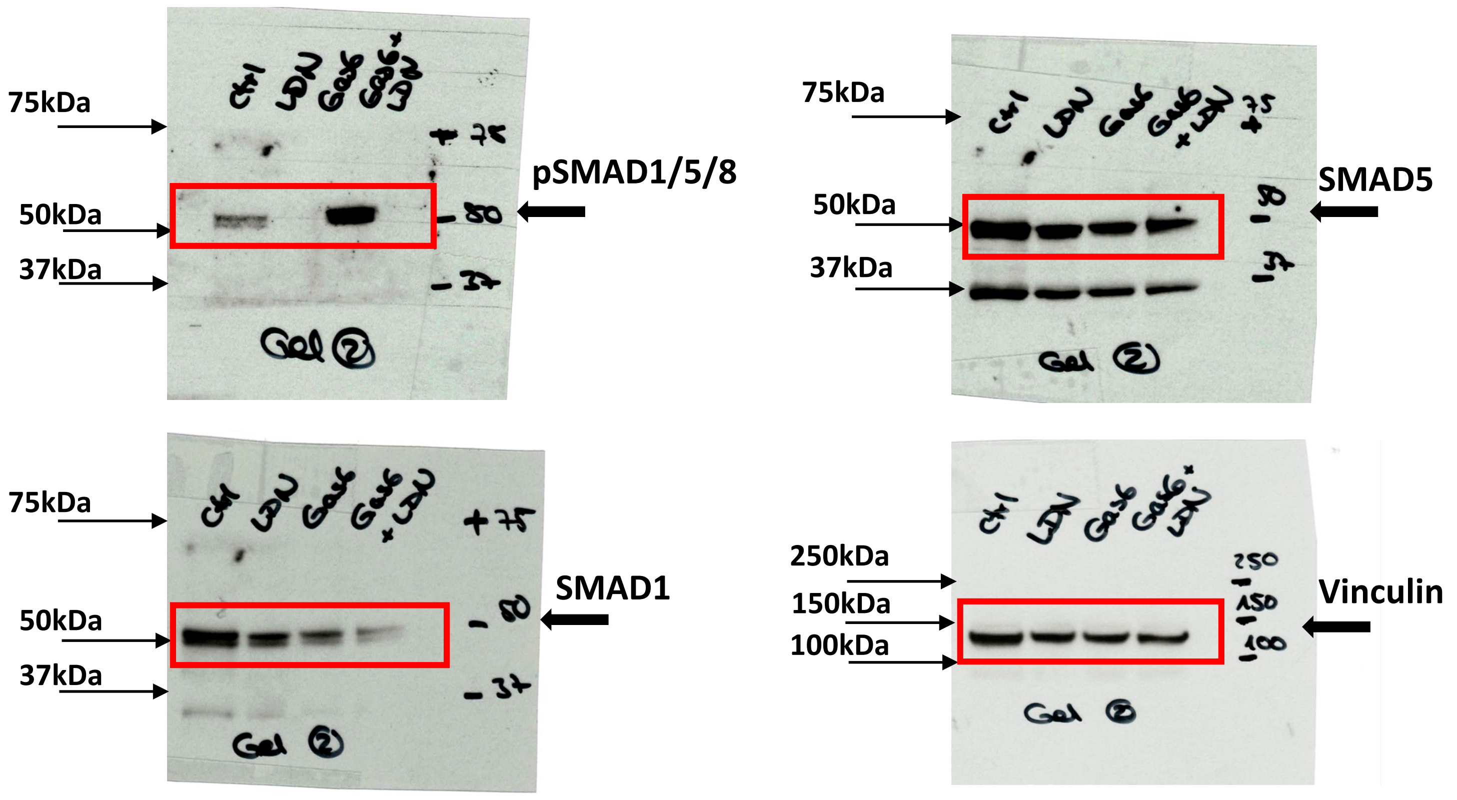
75kDa
50kDa
37kDa
pSMAD1/5/8
Gel 2
75kDa
50kDa
37kDa
SMAD5
Gel 2
75kDa
50kDa
37kDa
SMAD1
Gel 2
250kDa
150kDa
100kDa
Vinculin
Gel 2

Figure 7

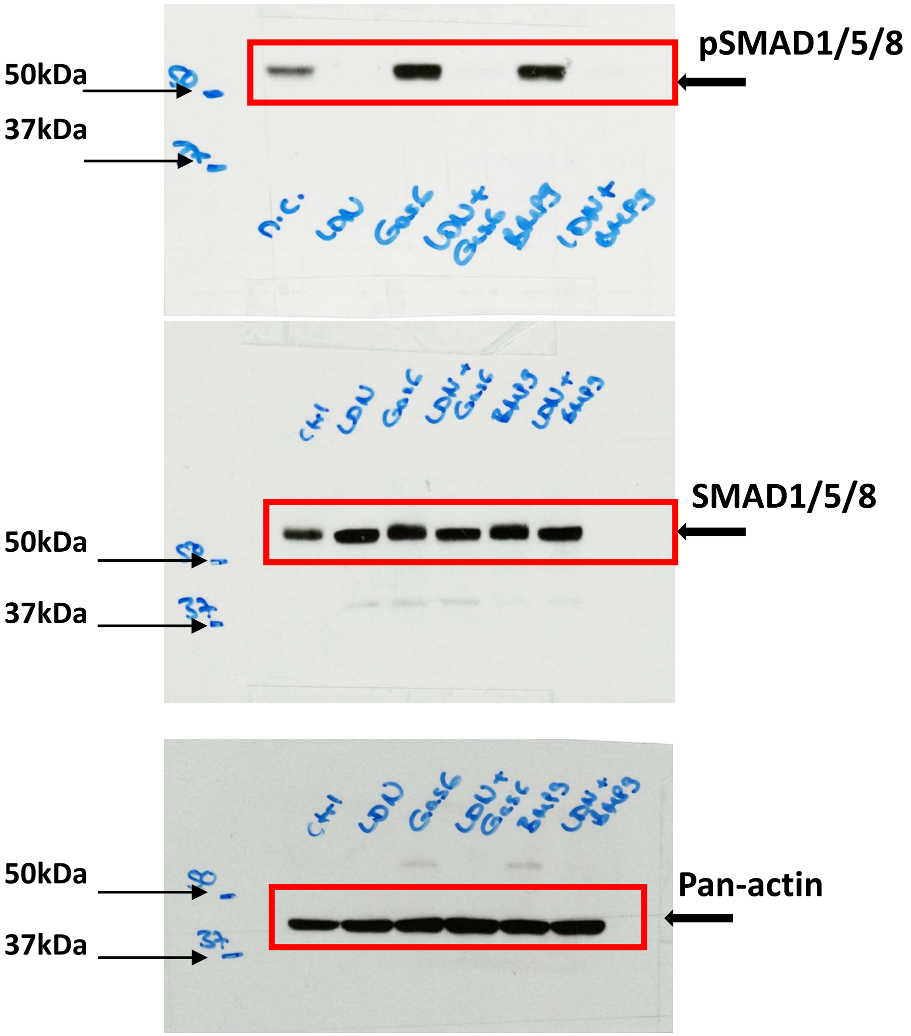
pSMAD1/5/8
50kDa
37kDa
SMAD1/5/8
50kDa
37kDa
Pan-actin
50kDa
37kDa

Figure 7

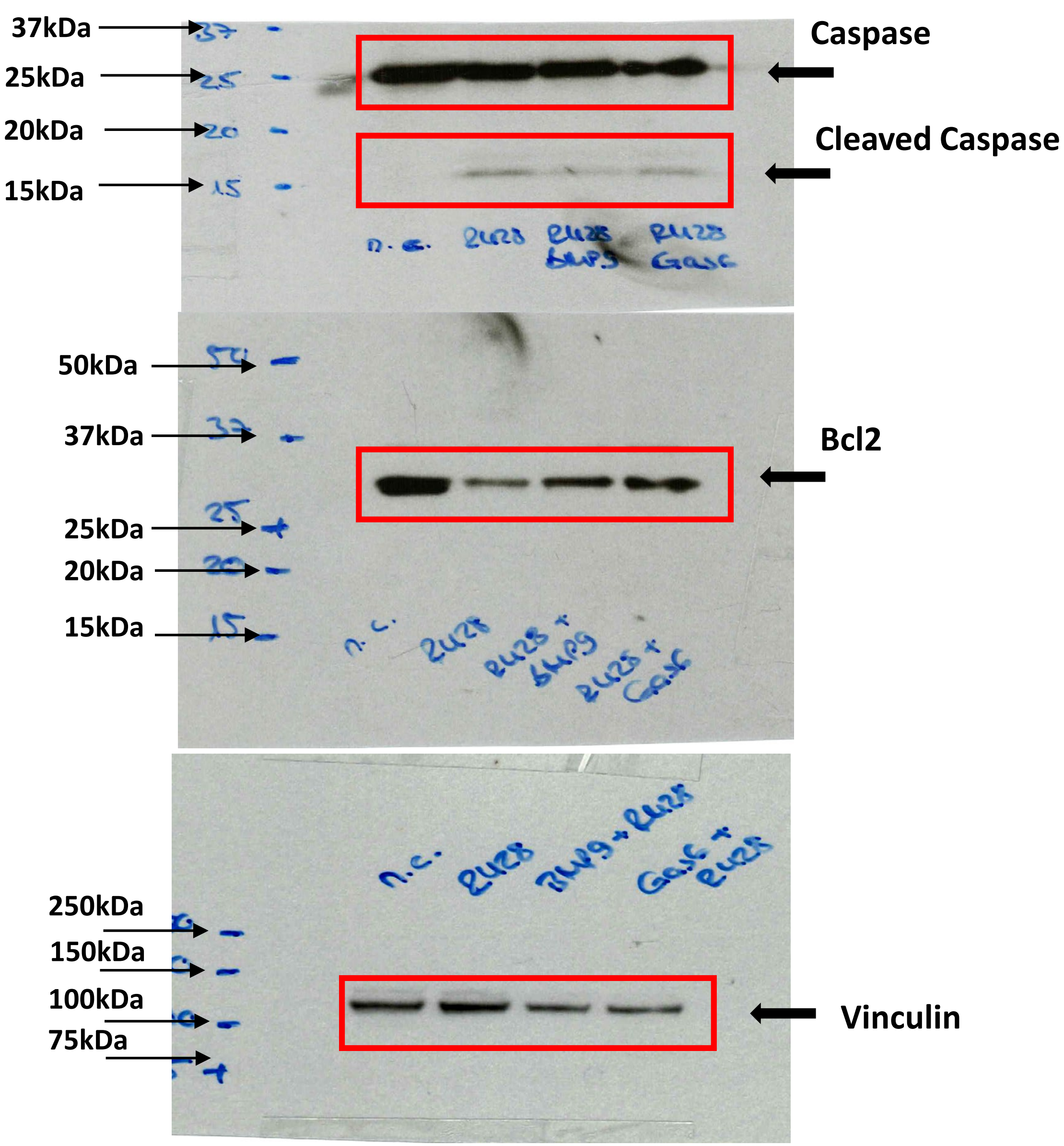
37kDa
25kDa
20kDa
15kDa
Caspase
Cleaved Caspase
50kDa
37kDa
25kDa
20kDa
15kDa
Bcl2
250kDa
150kDa
100kDa
75kDa
Vinculin

Figure 8

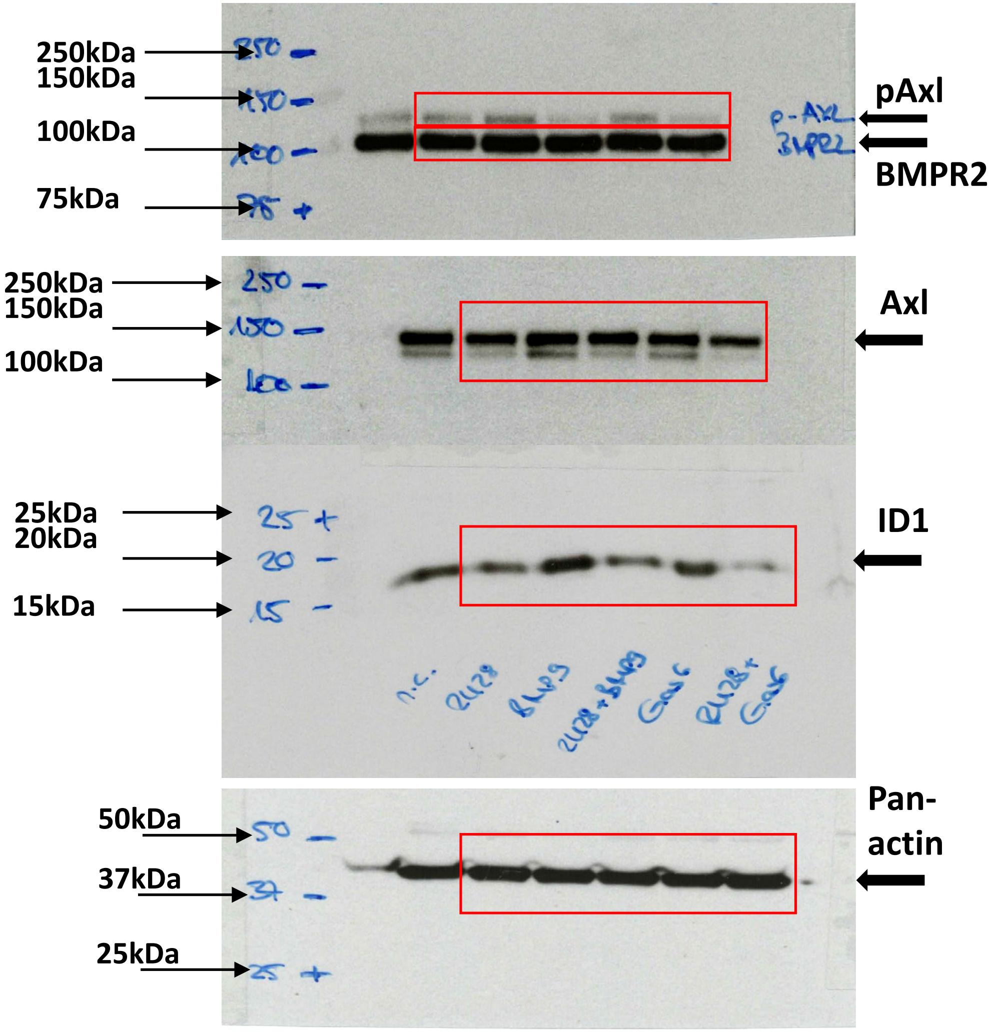

250kDa
150kDa
100kDa
75kDa
pAxl
BMPR2
250kDa
150kDa
100kDa
Axl
25kDa
20kDa
15kDa
ID1
50kDa
37kDa
25kDa
Pan-
actin

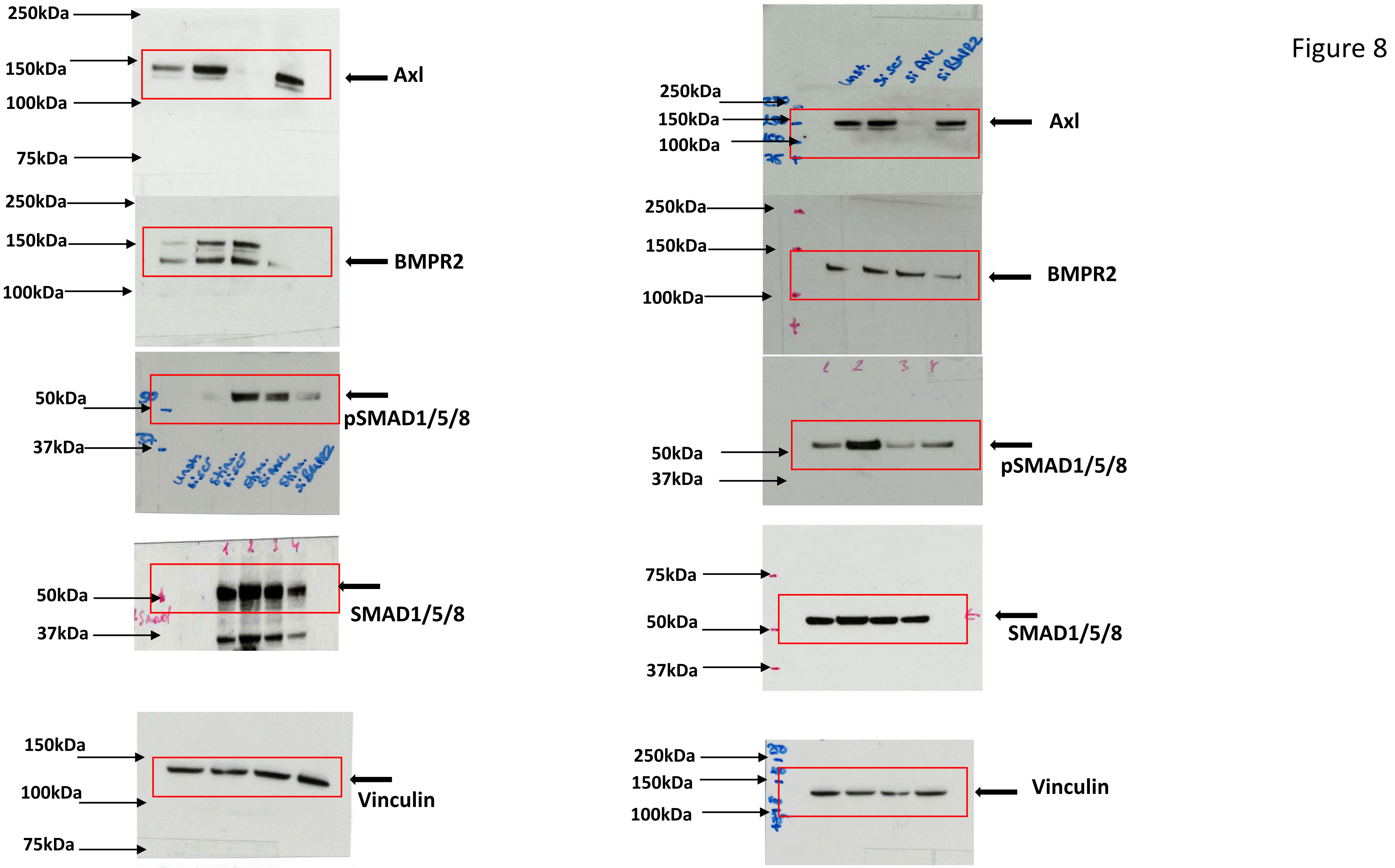
Figure 8
250kDa
150kDa
100kDa
75kDa
Axl
BMPR2
50kDa
37kDa
pSMAD1/5/8
SMAD1/5/8
Vinculin

Figure 9

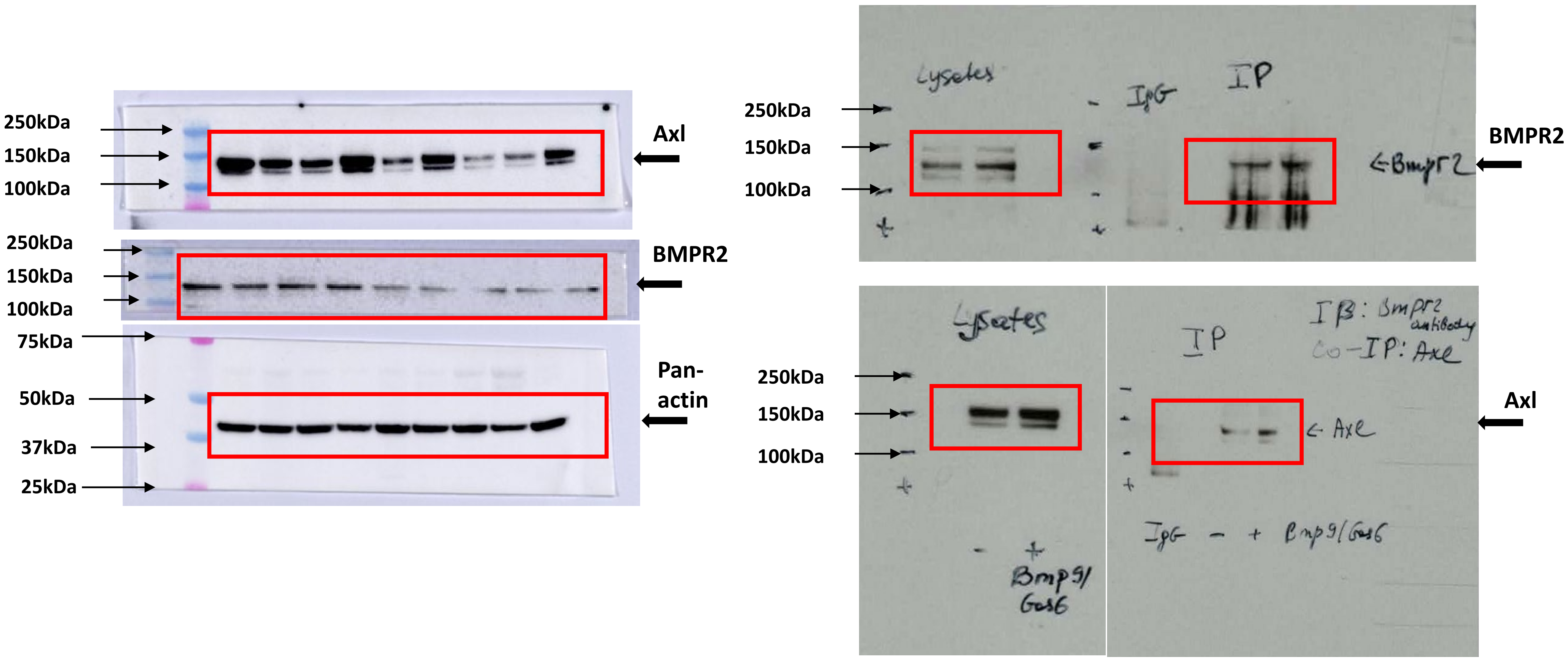
250kDa
150kDa
100kDa
Axl
250kDa
150kDa
100kDa
BMPR2
75kDa
50kDa
37kDa
25kDa
Pan-actin
Lysates
IgG
IP
250kDa
150kDa
100kDa
BMPR2
← Bmpr2
Lysates
IP
IB: Bmpr2 antibody
Co-IP: Axl
250kDa
150kDa
100kDa
← Axl
Axl
IgG − + Bmp9/Gas6
− + Bmp9/Gas6

Supplementary Figure 1

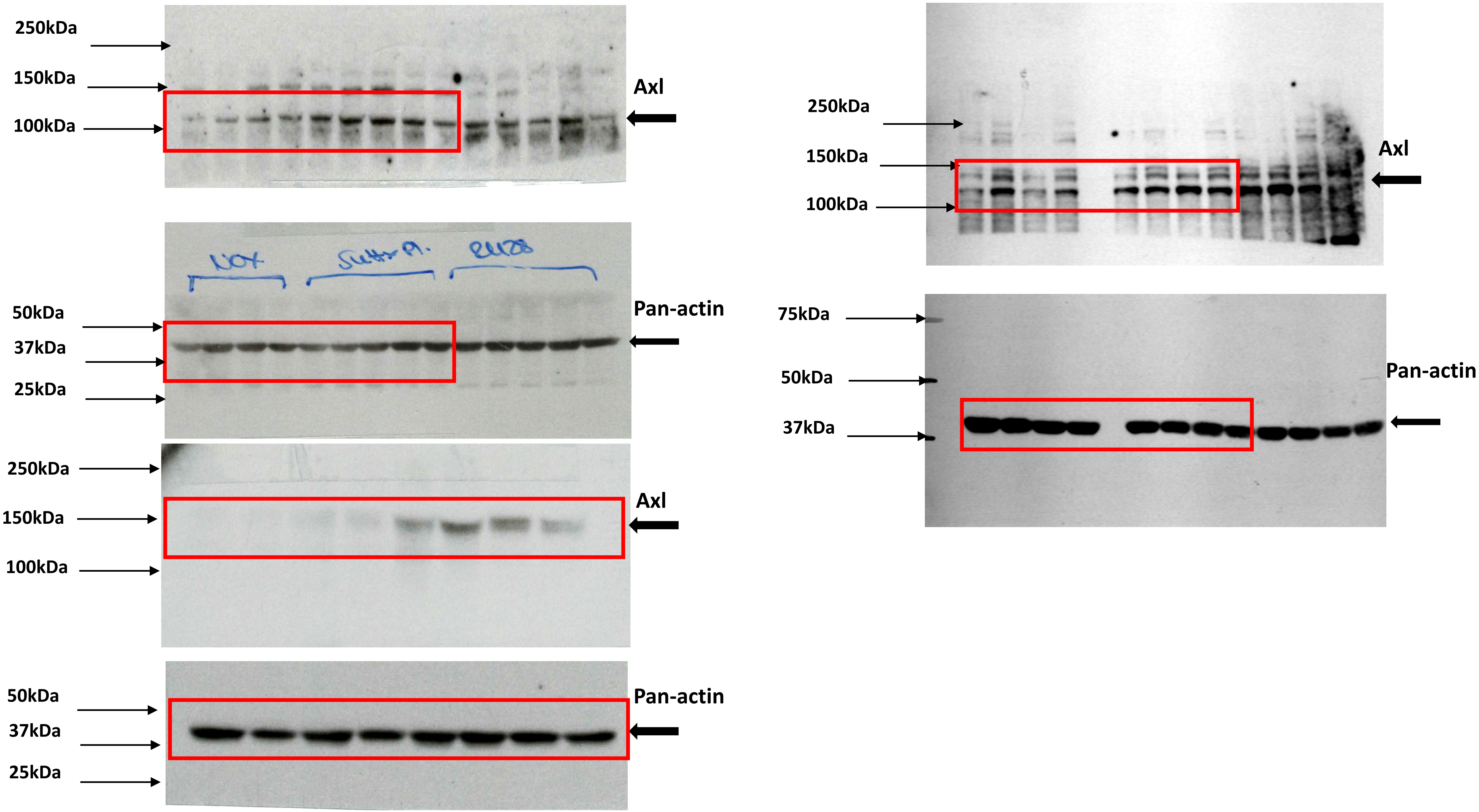

Supplementary Figure 1

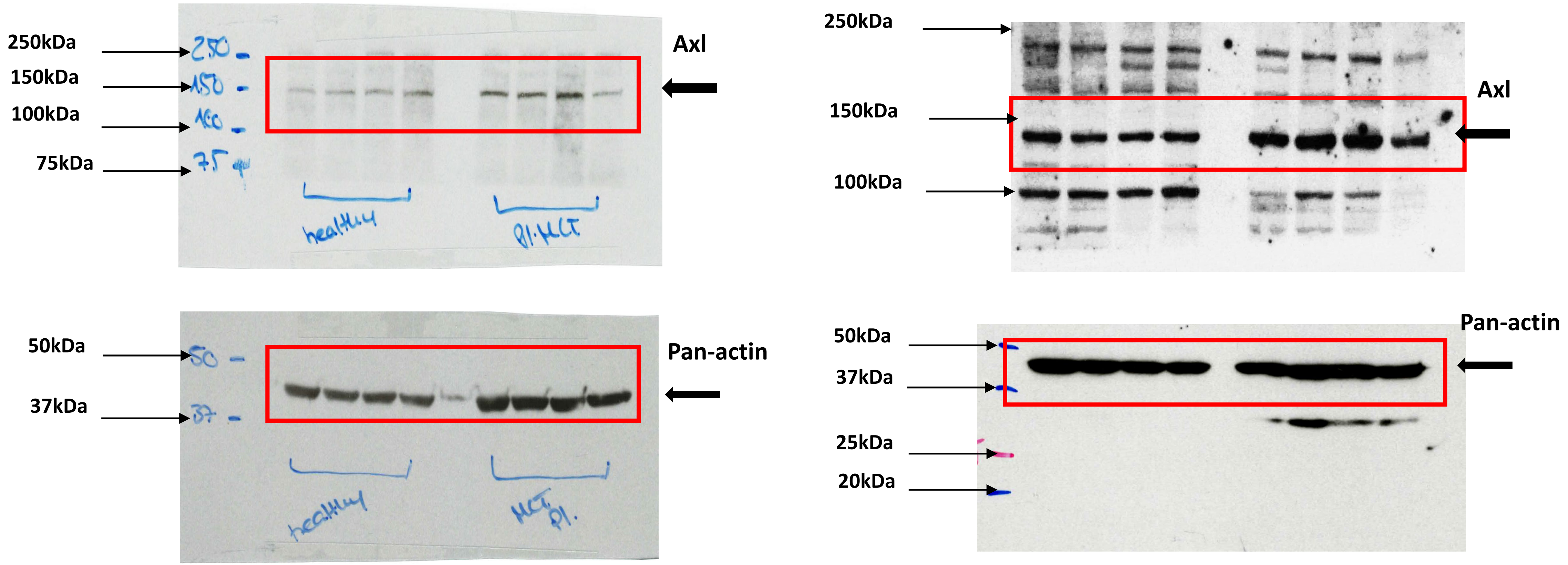

Supplementary Figure 1

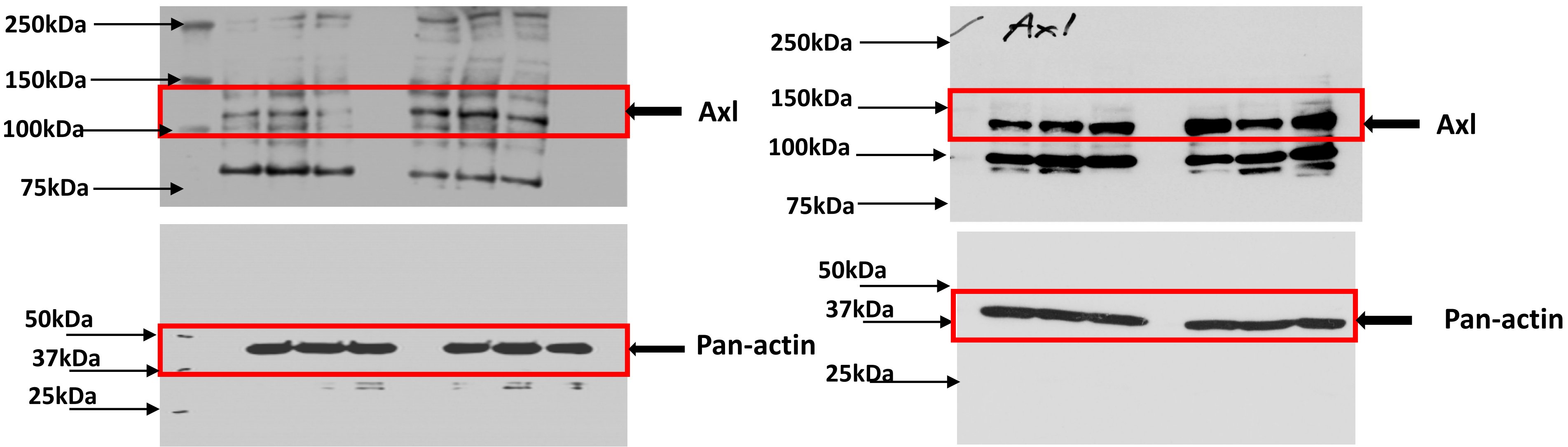

Supplementary Figure 3

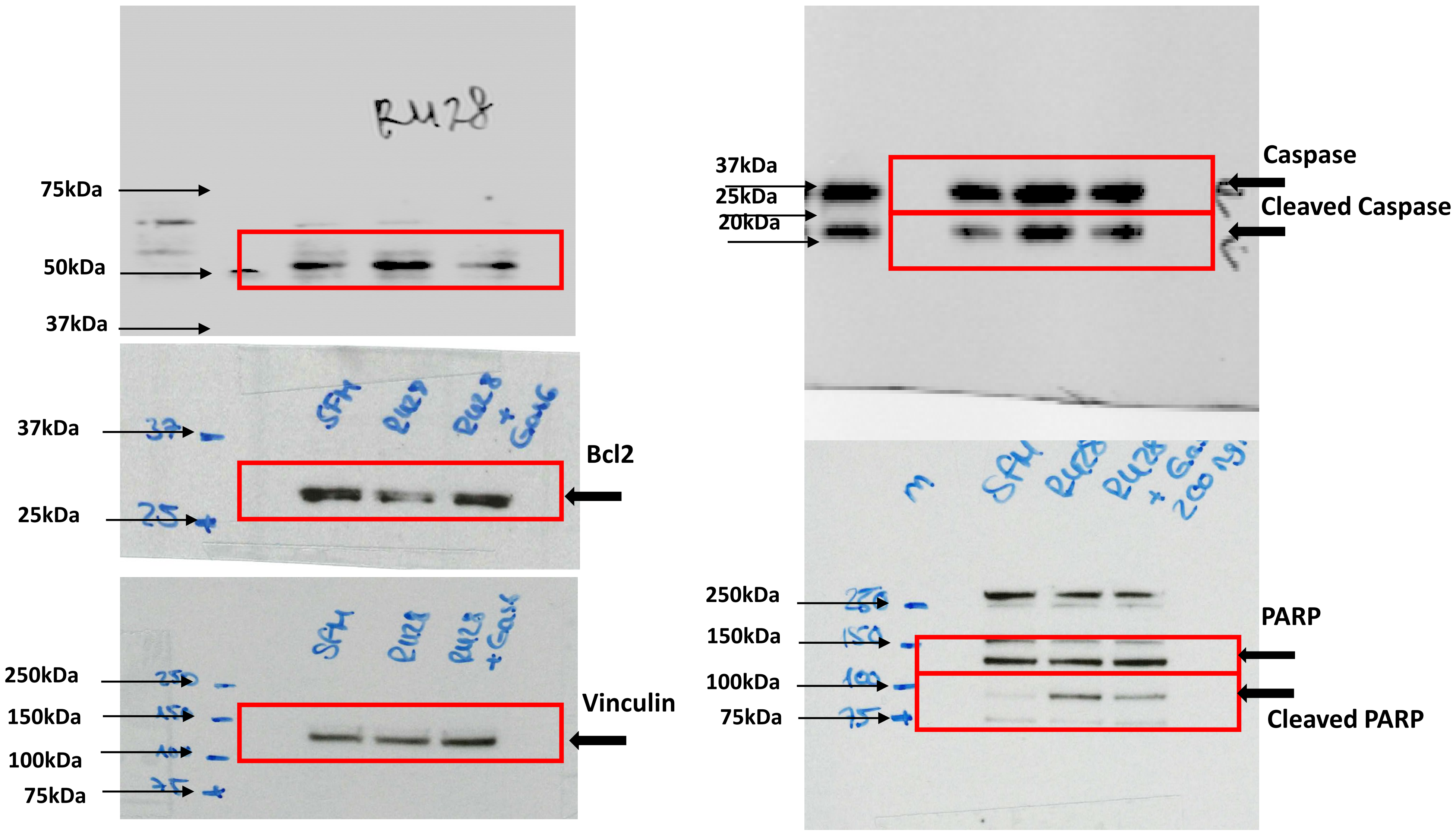

Supplementary Figure 4

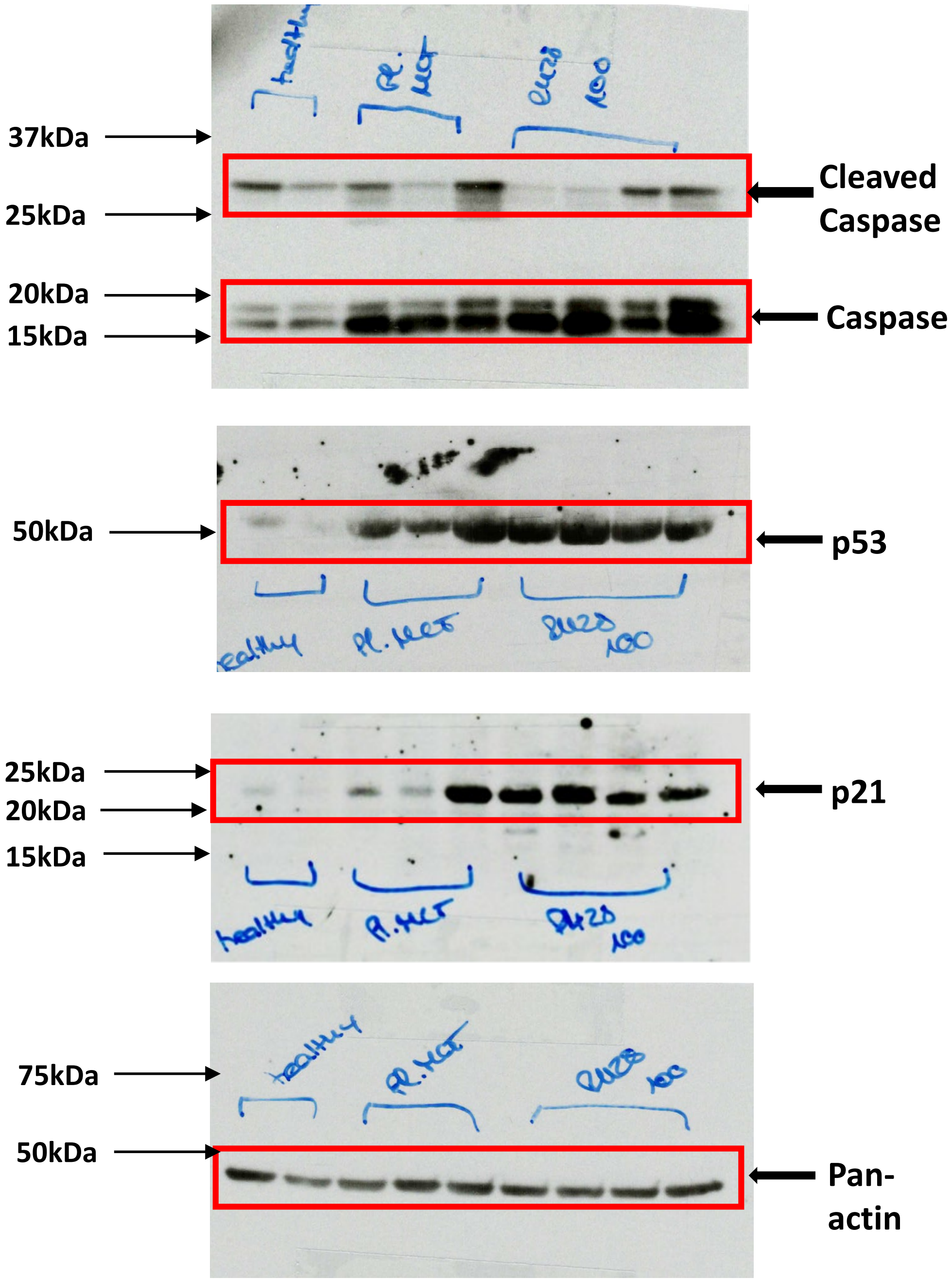

Supplementary Figure 5

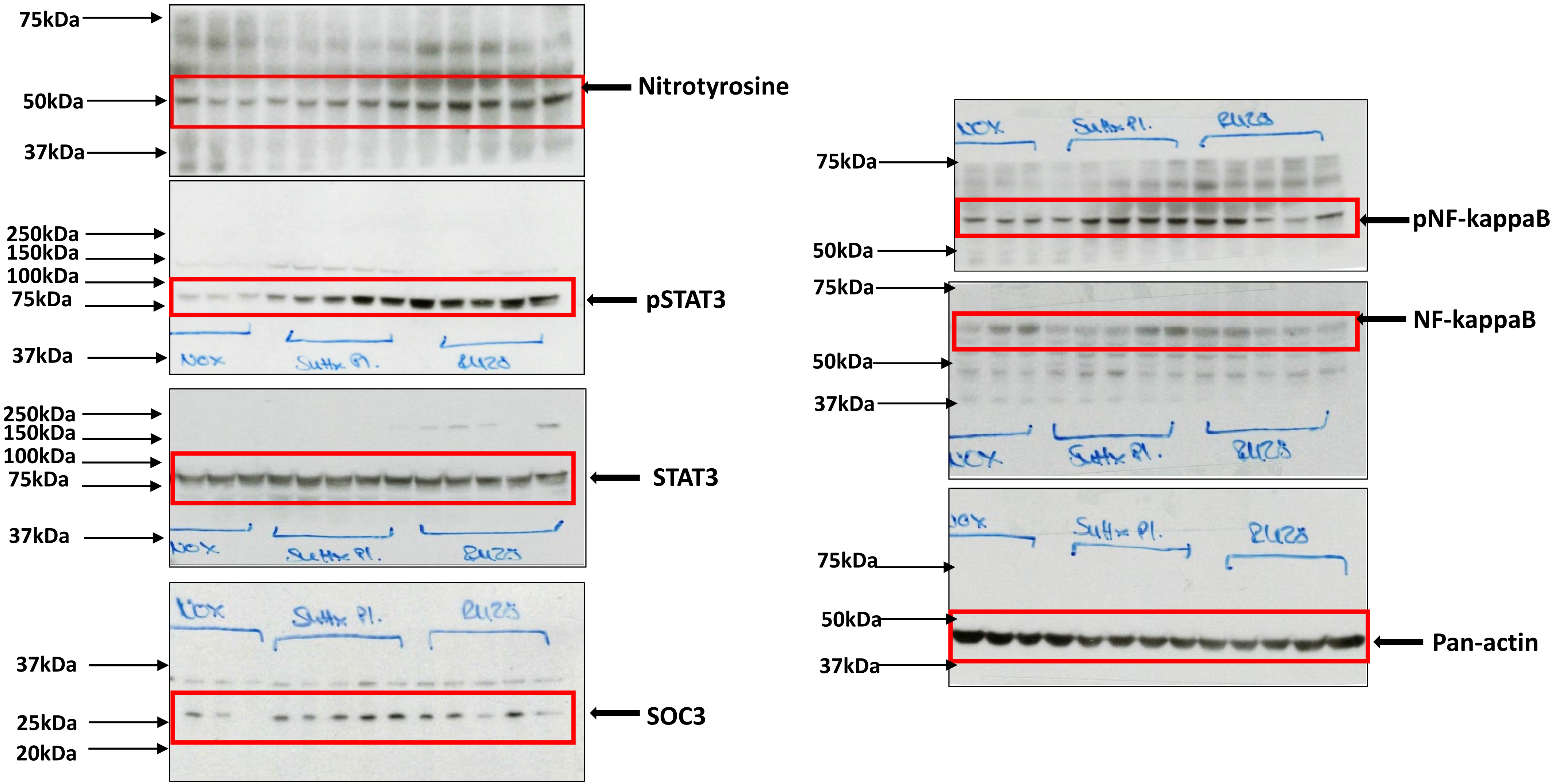

Supplementary Figure 6

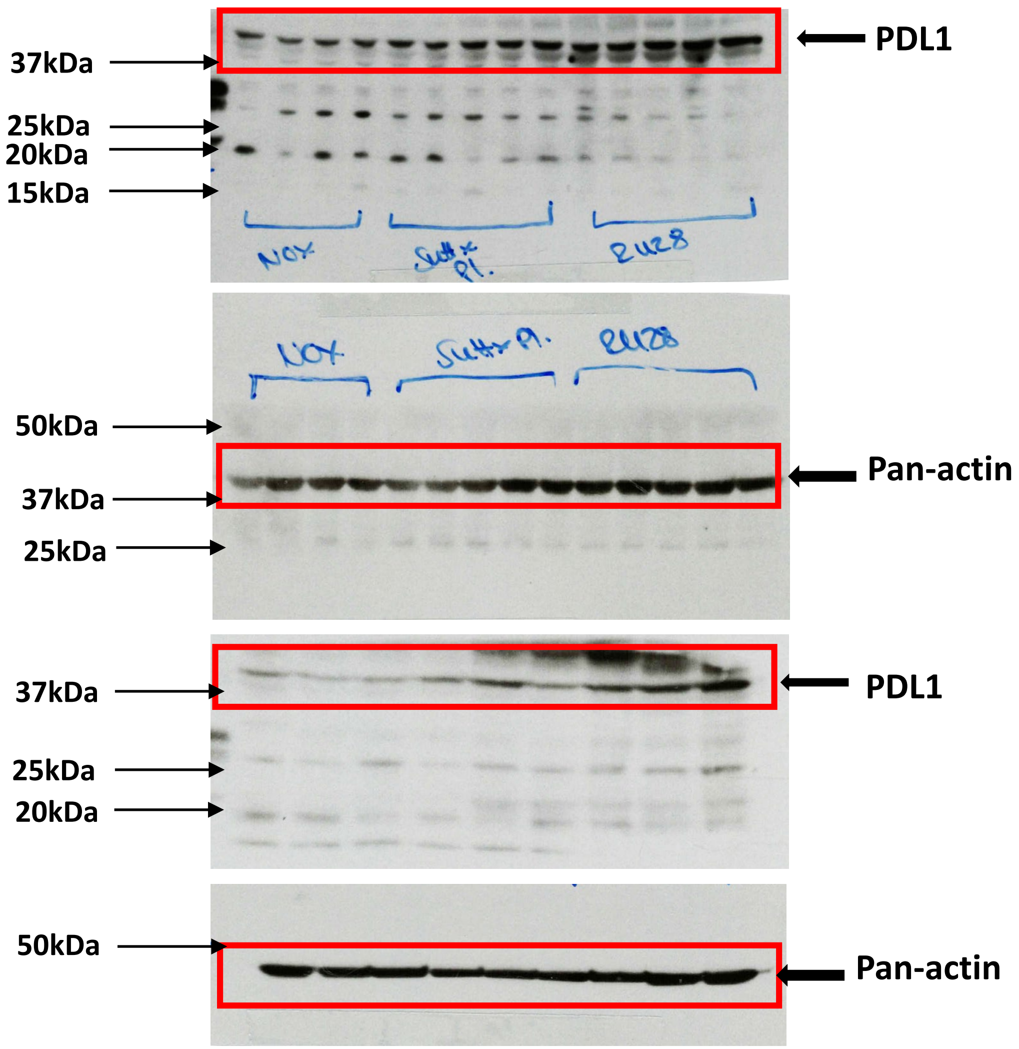

Supplementary Figure 7

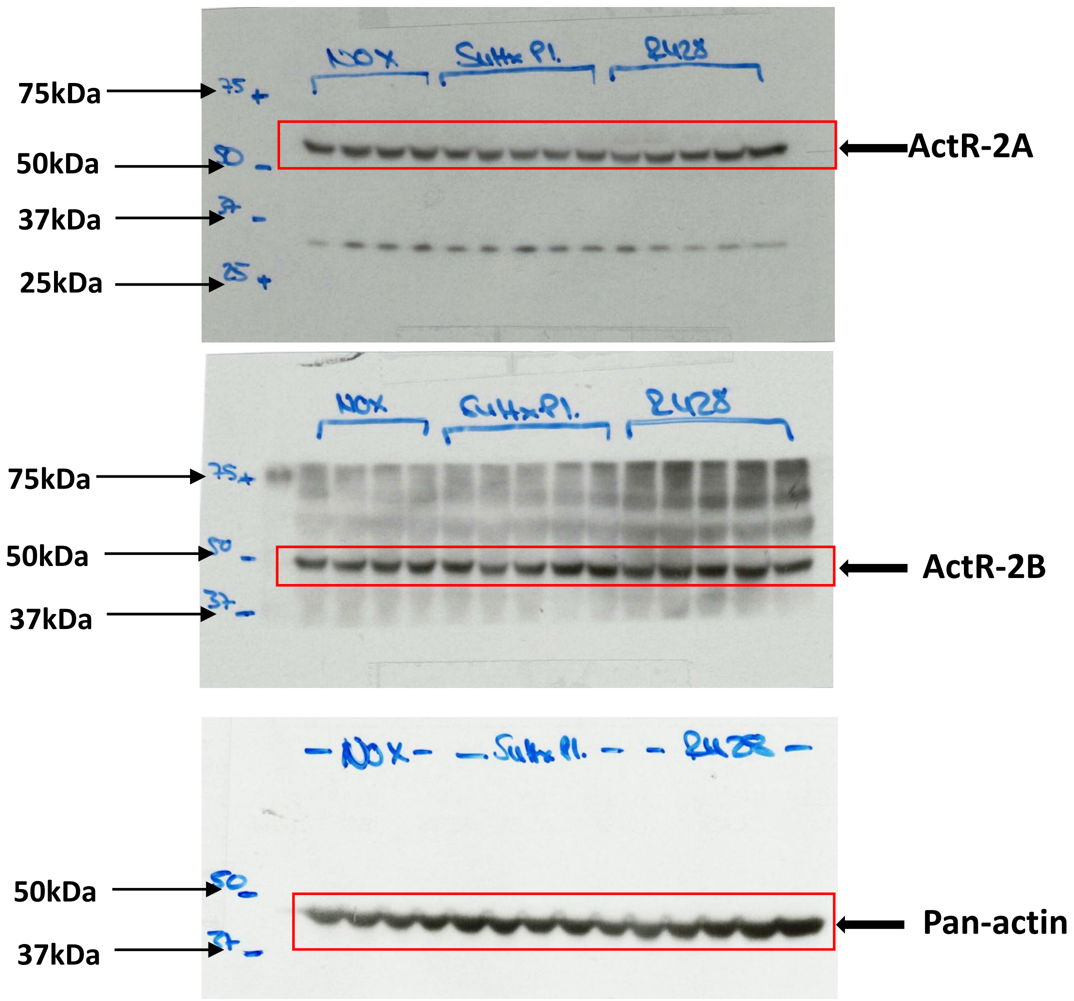

Supplementary Figure 8

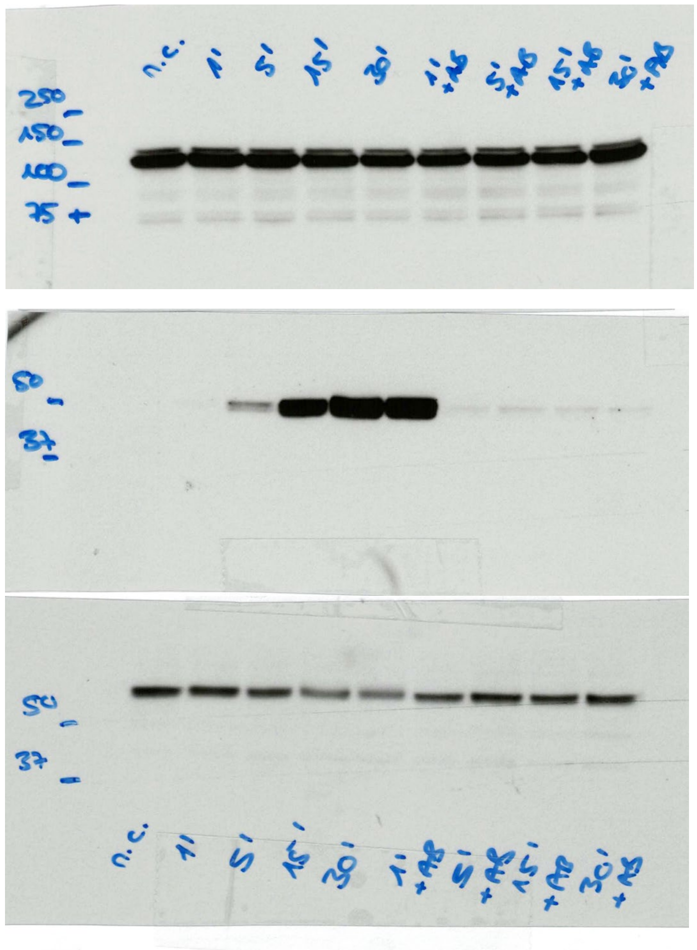

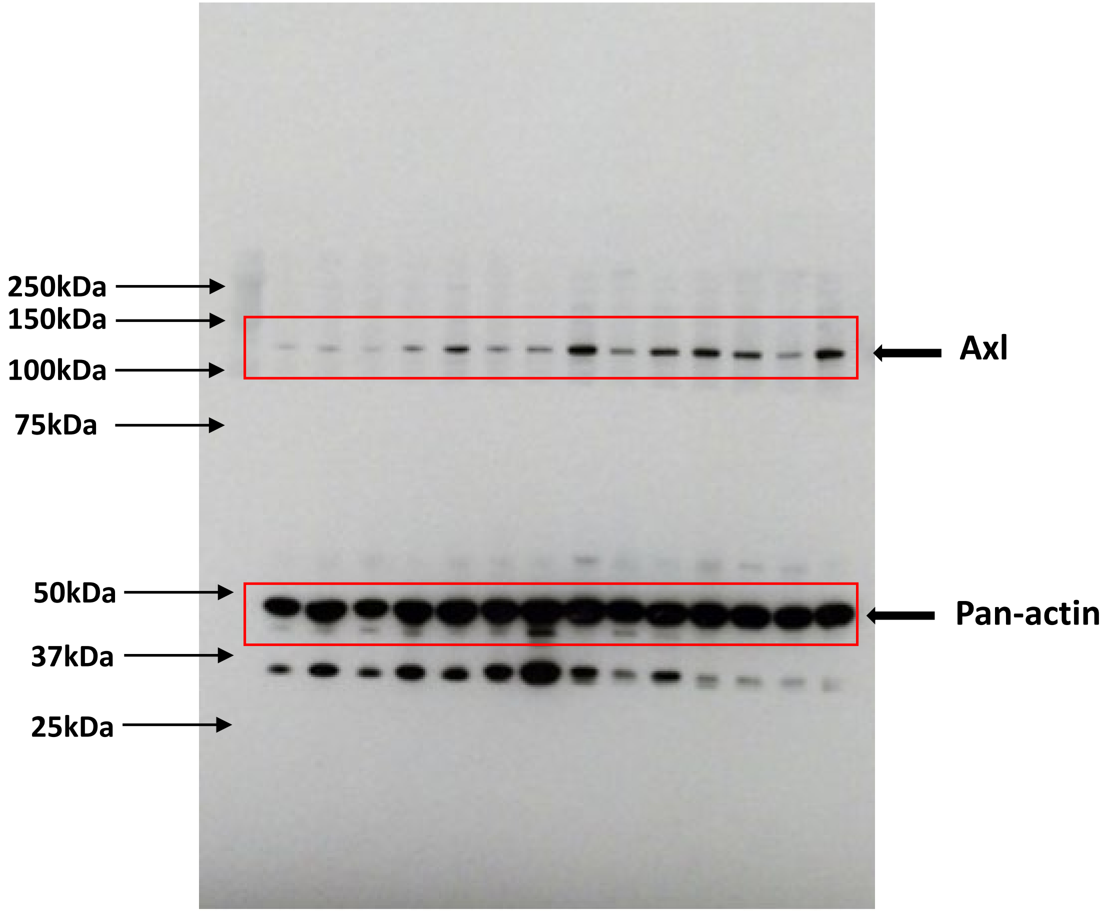

Supplementary Figure 8

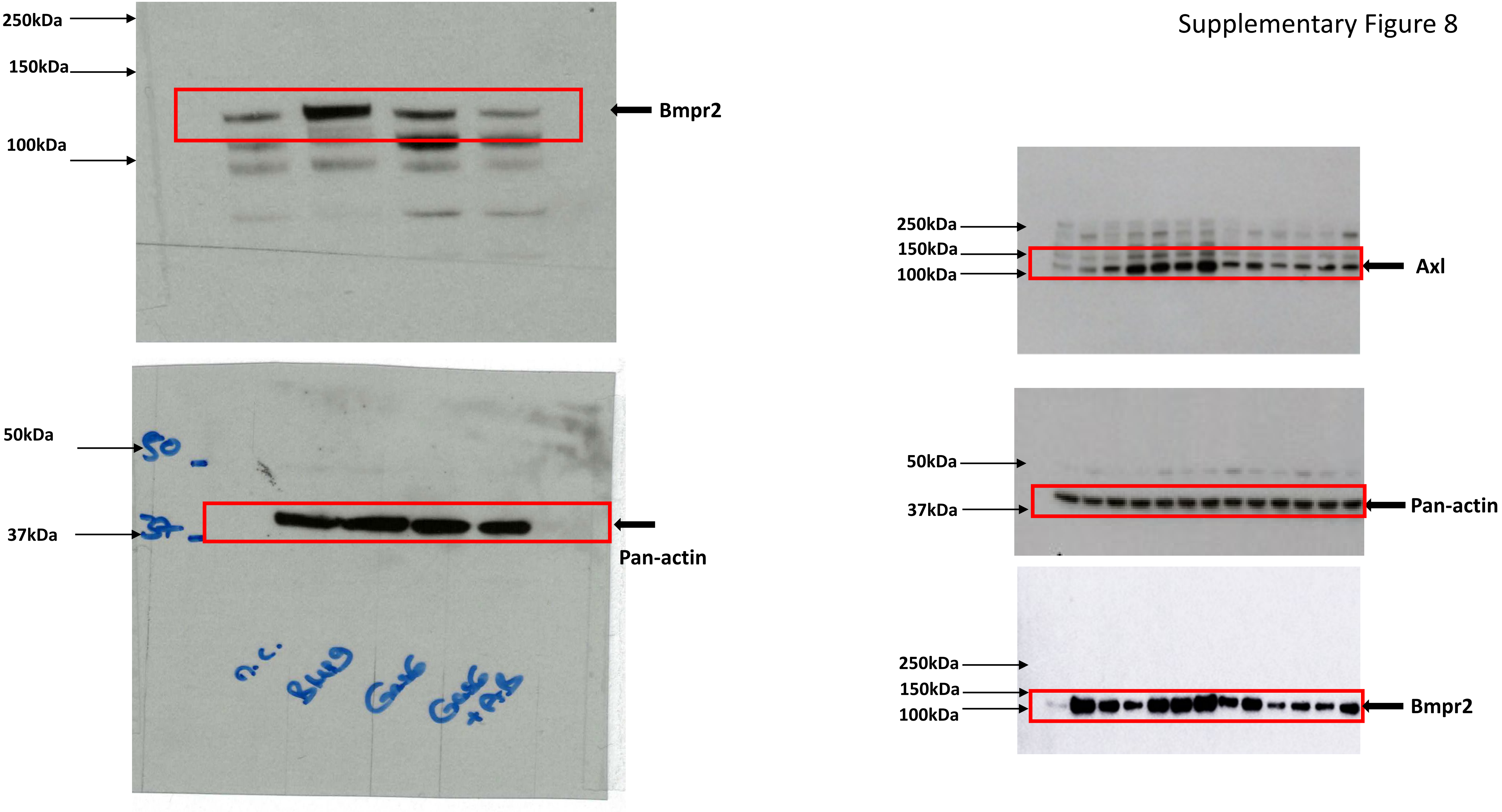

Supplement: Supplementary file 5 — Supplementary Data 2. [file 42003_2021_2531_MOESM5_ESM.pdf]
