# Supplementary material for: Deficiency of Axl aggravates pulmonary arterial hypertension via BMPR2
Source: Commun Biol. 2021 Aug 24;4:1002. doi: 10.1038/s42003-021-02531-1 (PMC8385080; doi:10.1038/s42003-021-02531-1)
Supplement: Supplementary file 6 — Reporting summary. [file 42003_2021_2531_MOESM6_ESM.pdf]

nature research

Corresponding author(s): Ralph Schermuly (COMMSBIO-20-2465-T)

Last updated by author(s): Sep 10, 2020

Reporting Summary

Nature Research wishes to improve the reproducibility of the work that we publish. This form provides structure for consistency and transparency in reporting. For further information on Nature Research policies, see our Editorial Policies and the Editorial Policy Checklist.

Statistics

For all statistical analyses, confirm that the following items are present in the figure legend, table legend, main text, or Methods section.

n/a	Confirmed	
☐	☒	The exact sample size (*n*) for each experimental group/condition, given as a discrete number and unit of measurement
☐	☒	A statement on whether measurements were taken from distinct samples or whether the same sample was measured repeatedly
☐	☒	The statistical test(s) used AND whether they are one- or two-sided<br>*Only common tests should be described solely by name; describe more complex techniques in the Methods section.*
☐	☒	A description of all covariates tested
☐	☒	A description of any assumptions or corrections, such as tests of normality and adjustment for multiple comparisons
☐	☒	A full description of the statistical parameters including central tendency (e.g. means) or other basic estimates (e.g. regression coefficient) AND variation (e.g. standard deviation) or associated estimates of uncertainty (e.g. confidence intervals)
☐	☒	For null hypothesis testing, the test statistic (e.g. *F*, *t*, *r*) with confidence intervals, effect sizes, degrees of freedom and *P* value noted<br>*Give P values as exact values whenever suitable.*
☒	☐	For Bayesian analysis, information on the choice of priors and Markov chain Monte Carlo settings
☒	☐	For hierarchical and complex designs, identification of the appropriate level for tests and full reporting of outcomes
☒	☐	Estimates of effect sizes (e.g. Cohen's *d*, Pearson's *r*), indicating how they were calculated

Our web collection on statistics for biologists contains articles on many of the points above.

Software and code

Policy information about availability of computer code

Data collection	*Provide a description of all commercial, open source and custom code used to collect the data in this study, specifying the version used OR state that no software was used.*
Data analysis	*Provide a description of all commercial, open source and custom code used to analyse the data in this study, specifying the version used OR state that no software was used.*

For manuscripts utilizing custom algorithms or software that are central to the research but not yet described in published literature, software must be made available to editors and reviewers. We strongly encourage code deposition in a community repository (e.g. GitHub). See the Nature Research guidelines for submitting code & software for further information.

Data

Policy information about availability of data

All manuscripts must include a data availability statement. This statement should provide the following information, where applicable:

- Accession codes, unique identifiers, or web links for publicly available datasets
- A list of figures that have associated raw data
- A description of any restrictions on data availability

The data underlying all findings of this study are available from the corresponding author upon reasonable request and are provided as a separate source data file.

Field-specific reporting

Please select the one below that is the best fit for your research. If you are not sure, read the appropriate sections before making your selection.

- [x] Life sciences
- [ ] Behavioural & social sciences
- [ ] Ecological, evolutionary & environmental sciences

For a reference copy of the document with all sections, see nature.com/documents/nr-reporting-summary-flat.pdf

Life sciences study design

All studies must disclose on these points even when the disclosure is negative.

Sample size	Sample sizes were determined depending on the experimental settings and availability of the specimens. Due to limitations in the availability and the amount of healthy and diseased human lung tissues and subsequently sections and isolated primary cells (hPASMCs, hBOECs, hPAECs) lower numbers of biospecimens and cells are present. For in vivo experiments, we were restricted to certain sample sizes due to our animal proposal. All of our findings were only reported to be conclusive and successful if statistical significance was obtained. All numbers and the type of replicates (i.e. biological versus technical) can be found in the source data file.
Data exclusions	Data points were excluded only in very few cases, if they could be identified as the outliers due to technical failures of experiments (indicated in the source data file).
Replication	All our findings were clearly reproducible as indicated by using multiple biological as well as technical replicates and independently repeated measurements.
Randomization	For the in vivo study, diseased animals (MCT- or Su5416-injected) were randomly allocated into two groups i.e. placebo (MCT ; Su/Hox) or treatment (MCT + R428; Su/Hox + R428).
Blinding	In vitro studies were not performed in a blinded fashion due to the high risk of confusion in handling of the samples by different experimenters. For the quantification of IF and IHC stainings, the investigator/s were blinded to ensure an unbiased interpretation of the results

Reporting for specific materials, systems and methods

We require information from authors about some types of materials, experimental systems and methods used in many studies. Here, indicate whether each material, system or method listed is relevant to your study. If you are not sure if a list item applies to your research, read the appropriate section before selecting a response.

Materials & experimental systems

n/a	Involved in the study	
[ ]	[x]	Antibodies
[ ]	[x]	Eukaryotic cell lines
[x]	[ ]	Palaeontology and archaeology
[ ]	[x]	Animals and other organisms
[x]	[ ]	Human research participants
[x]	[ ]	Clinical data
[x]	[ ]	Dual use research of concern

Methods

n/a	Involved in the study	
[x]	[ ]	ChIP-seq
[x]	[ ]	Flow cytometry
[x]	[ ]	MRI-based neuroimaging

Antibodies

Antibodies used

Antibodies used for Western Blotting:
ActR-2a Santa Cruz Biotechnology, Dallas, US sc-515826
ActR-2b Santa Cruz Biotechnology, Dallas, US sc-134245
AXL (C89E7) Cell Signaling Technology, Inc., Danvers, US #8661S
BCL-2 (C-2) Santa Cruz Biotechnology, Dallas, US sc-7382
BCL-2 (N-19) Santa Cruz Biotechnology, Dallas, US sc-492
BMPR2 Abbexa Ltd, Cambridge, UK abx007889
BMPR2 (E-1) Santa Cruz Biotechnology, Dallas, US sc-393304
BMPR2 BD Biosciences, Sna Jose, CA, US 612292
Caspase-3 Cell Signaling Technology, Inc., Danvers, US #9662S
Cleaved Caspase-3 (Asp175) Cell Signaling Technology, Inc., Danvers, US #9661S
Cyclin D1 (H-295) Santa Cruz Biotechnology, Dallas, US sc-753
Gas6 R&D Systems, Minneapolis, US AF885
ID1 (B-8) Santa Cruz Biotechnology, Dallas, US sc-133104

NFκB p65 (F-6) Santa Cruz Biotechnology, Dallas, US sc-8008
Nitrotyrosine (1A6) Merck Millipore, Burlington, MA, US 06-284
p21 (F-5) Santa Cruz Biotechnology, Dallas, US sc-6246
p27 (F-8) Santa Cruz Biotechnology, Dalls, US sc-1641
p53 (1C12) Cell Signaling Technology, Inc., Danvers, US #2524S
Pan-Actin Cell Signaling Technology, Inc., Danvers, US #4968S
PD-L1 (CD274) Abbexa Ltd, Cambridge, UK abx001385
pAXL (Y779) R&D Systems, Minneapolis, US MAB6965
pSmad 1/5/9 Cell Signaling Technology, Inc., Danvers, US #13820S
pSTAT3 (S727) Cell Signaling Technology, Inc., Danvers, US #9134S
pNFκB p65 (S536) Santa Cruz Biotechnology, Dallas, US sc-136548
Smad 1/5/8 (N-18) Santa Cruz Biotechnology, Dallas, US sc-6031-R
pSmad2 Cell Signalling Technology, Inc., Danvers, US #3108
pSmad3 Cell Signalling Technology, Inc., Danvers US #9520
Smad2/3 Cell Siganlling Technology, Inc., Danvers US #3102
Smad3 Cell Signalling Technology, Inc., Danvers US#9523
SOCS3 Cell Signaling Technology, Inc., Danvers, US #2923
STAT3 (79D7) Cell Signaling Technology, Inc., Danvers, US #4904S

Antibodies used for IF/IH:
AXL (C89E7), Cell Signaling Technology, Inc., Danvers, US, #8661
AXL R&D Systems, Minneapolis, US, AF854
CD68 (clone ED1) , Bio-Rad Laboratories GmbH, Feldkirchen, Germany, MCA341GA
Isolectin GS-IB4 (from Griffonia simplicifolia), Thermo Fisher Scientific, Waltham, US, I21411
PCNA, Cell Signaling Technology, Danvers, MA, US #2586
α-SMA, Sigma-Aldrich (now Merck), Darmstadt, Germany, A2547
vWF, Dako (now Agilent), Hamburg, Germany, A0082

Validation

All antibodies were carefully selected for the desired application and utilized according to the manufacturer´s instructions. Validation statements can be found on the suppliers website and related positive/negative controls were performed in parallel in our study. To establish IHC and IF stainings for Axl several antibodies from different companies have been tested (Cell Signalling, &D systems, BIOSS, Novus Biologicals).
In total, we tested multiple antibodies for all the proteins from the following companies: Abcam, Abbexa,Biorbyt, Bio-Rad Laboratories, Cell Signaling, DAKO, Merck Millipore, R&D systems, Santa Cruz Biotechnology, Sigma Aldrich and Thermo Fisher Scientific. In our study we used the best working ones giving a specific signal; those are listed above.

Eukaryotic cell lines

Policy information about cell lines

Cell line source(s)

Only isolated primary cells were used which were provided from Lonza Ltd. (Basel, Switzerland) as well as from the UGMLC Giessen Biobank. Primary arterial endothelial cells (hPAECs) were provided from PromoCell (Heidelberg, Germany), as well as from Service de Pneumologie et Soins Intensifs Respiratoires (France)
- Human pulmonary arterial smooth muscle cells (hPASMCs)
- Human pulmonary arterial endothelial cells (hPAECs)

Authentication

Cells obtained from Lonza Ltd. (Basel, Switzerland) were authenticated by the manufacturer . Cells provided by the UGMLC Giessen Biobank were characterized by immunofluorescence staining positive for SMA and negative for EC-specific vWF or Fibroblast-specific Vimentin.

Mycoplasma contamination

All cells used for the experiments were tested for mycoplasma contamination by PCR prior to our studies.

Commonly misidentified lines
(See ICLAC register)

Name any commonly misidentified cell lines used in the study and provide a rationale for their use.

Animals and other organisms

Policy information about studies involving animals; ARRIVE guidelines recommended for reporting animal research

Laboratory animals

MCT model: Sprague-Dawley rats, male, 300-350 g body weight
Su/Hox model: Wistar-Kyoto rats, male, 300-350 g body weight
Body weight was chosen as specific parameter for proper dose calculation and drug administration.

Wild animals

No wild animals were used in this study.

Field-collected samples

The study did not involve the samples collected from the field.

Ethics oversight	Both, the University Animal Care Committee and the federal authorities for animal research of the Regierungspräsidium Giessen and Darmstadt (Hesse, Germany) approved the study protocol (approval numbers GI 20/10 Nr. G 50/2016 and GI 20/10 Nr.33/3013).

Note that full information on the approval of the study protocol must also be provided in the manuscript.
